# Comprehensive Analysis and Validation of Solute Carrier Family 25 (SLC25) and Its Correlation with Immune Infiltration in Pan-Cancer

**DOI:** 10.1155/2022/4009354

**Published:** 2022-10-08

**Authors:** Ao-ran Liu, Ying-nan Liu, Shi-xuan Shen, Li-rong Yan, Zhi Lv, Han-xi Ding, Ang Wang, Yuan Yuan, Qian Xu

**Affiliations:** ^1^Tumor Etiology and Screening Department of Cancer Institute and General Surgery, The First Hospital of China Medical University, No. 155 North NanjingBei Street, Heping District, Shenyang, 110001 Liaoning, China; ^2^Key Laboratory of Cancer Etiology and Prevention in Liaoning Education Department, The First Hospital of China Medical University, Shenyang 110001, China; ^3^Key Laboratory of GI Cancer Etiology and Prevention in Liaoning Province, The First Hospital of China Medical University, Shenyang 110001, China

## Abstract

As the largest gene family functioning in protein transport among human solute carriers, the SLC25 family (mitochondrial carrier family) can participate in development of cancer. However, a comprehensive exploration for the exactly roles of SLC family remains lacking. In the present study, a total of 15 functional SLC25 family genes were retrieved from all current publications. And multidimensional analyses were systematically performed based on the transcriptome and genome data of SLC25 family from a variety of online databases for their expression, immune cell infiltration, and cancer prognosis. Validation by qPCR and immunohistochemistry were further conducted for the expression of partial SLC25 family members in some tumor tissue. We found that the SLC25 family had strong correlation with immune cells, such as macrophages M2, CD8^+^ T cell, CD4^+^ T cell memory activated, and memory resting. Among them, SLC25A6 was most correlated with Macrophage M1 in uveal melanoma (*r* = −0.68, *P* = 1.9*e* − 0.5). Expression of mRNA level showed that SLC25A4 was downregulated in stomach adenocarcinoma and colon adenocarcinoma. SLC25A7 was highly expressed in stomach adenocarcinoma and colon adenocarcinoma. SLC25A23 was decreased in colon adenocarcinoma. qPCR and immunohistochemistry validation results were consistent with our bioinformatics prediction. SLC25A8 was associated with the prognosis of cancer. All these findings suggested that the SLC25 family might affects the immune microenvironment of the cancer and then had the potential to be predictive biomarkers for early diagnosis and prognosis as well as novel targets for individualized treatment of cancer.

## 1. Introduction

As the center of energy metabolism, mitochondria play vital roles in human physiological activities, and substantial material exchange occurs in mitochondrial membrane at all times. Mitochondrial membrane consists of outer mitochondrial membrane (OMM) and inner mitochondrial membrane (IMM). The permeability of OMM is relatively high, while IMM has a lower permeability with higher requirement for penetration. Only proteins with a molecular weight of less than 15 kD can pass through IMM [1]. Therefore, some carriers on IMM are essential for transmembrane transport to participate in important cellular activities, such as fat metabolism, oxidative phosphorylation, and biomacromolecule synthesis [2].

Cancer cell energy metabolism is a way that cancer cells will undergo glycolysis to produce energy even under aerobic conditions. This “aerobic glycolysis” and oxidative phosphorylation coordinate with each other to produce metabolism of symbiosis, which makes cancer cells and tumor microenvironment adaptable for metabolic reprogramming. However, in order to maintain effective oxidative phosphorylation, the IMM becomes impermeable, so IMM carriers must be required to transport energy metabolites to meet metabolic supply. The uptake of a large amount of glucose in the microenvironment by tumor cells can inhibit the mTOR activity of T cells and glycolytic ability. The lactic acid secreted by cancer cells undergoing glycolysis and aerobic phosphorylation in the mitochondria cannot only acidify the microenvironment but also polarize tumor-associated macrophages to M2 type to promote tumor growth.

The solute carrier protein 25 (SLC25) family, the largest gene transporter family, is a kind of membrane protein that controls various solutes in and out and thus participates in important physiological activities such as cell material transport, energy transmission, signal transduction, and nutrient metabolism. There are 53 family members discovered so far. These carrier proteins transport macromolecular solutes across IMM regardless of molecular size limitation and contribute in many cellular processes.

In recent years, knowledge for the function of SLC25 family has been increasingly enriched with continuous in-depth research. Lots of experiments have confirmed that SLC25s exert regulatory roles in all aspects of physiological process, pathological process, and disease progression [3, 4]. Numerous studies have indicated that the SLC25 family is associated with cancer and its metabolic reprogramming. For example, a study found that the expression of SLC25A10 could reprogram tumor metabolism, affect its growth, and enhance the sensitivity of tumor cells to chemotherapeutic drugs such as cisplatin, which was considered a new therapeutic target [5]. Therefore, the SLC25 family has the potential to be a novel tumor biomarker, which has profound significance for cancer diagnosis, treatment, and prevention [6].

**Figure 1:**
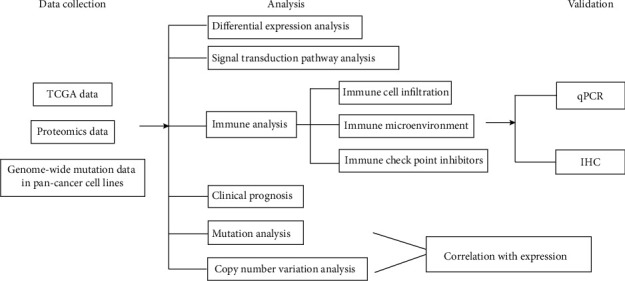
The flowchart of this study. We collected data from multiple databases and analyzed the multiomics profiles of SLC25s in pan-cancer. Finally, the prediction results were validated by qPCR and IHC with tissue samples.

Currently, however, the function of many SLC25 genes has not been elucidated yet, and specific mechanism needs to be explored for the influence of SLC25 family on tumor biological behaviors. According to the currently known functions of SLC25 family members, as mitochondrial carriers, the SLC25 family may participate in the occurrence and progression of cancer by affecting the immune microenvironment. Here, multidimensional analyses were systematically performed for the expression status (tissue mRNA, protein, and cell lines) of the SLC25 family from the perspective of pan-cancer based on a variety of online databases. And their association with immune cell infiltration and prognosis in pan-cancer was subsequently analyzed. Our study is aimed at providing theoretical basis for the potential of SLC25 family as tumor biomarkers and targets (Figure 1).

## 2. Materials and Methods

### 2.1. Data Collection

#### 2.1.1. Selection of SLC25 Family

A total of 53 SLC25 family genes were selected from published review articles (Table S1). However, based on the fact that some genes have no function or functions are not yet discovered, 15 genes of the SLC25 family with accurate functions were selected for analysis in our study. And they were manually converted into Ensembl gene IDs and HGNC symbols according to the Gene Cards (https://www.genecards.org/).

#### 2.1.2. Collection of TCGA Data

Information about 33 different types of cancer was collected from TCGA database (http://cancergenome.nih.gov/) (Table S2), including TPM (Transcripts Per Kilobase Million) expression, copy number variation, and mutation as well as clinical information (survival status, stage, grade, and survival time) downloaded from UCSC Xena (https://Xenabrowser.net/).

#### 2.1.3. Collection of Proteomics Data

The protein expression of SLC25 family was obtained from the Human Protein Atlas (https://www.proteinatlas.org/) containing 21 types of cancer and corresponding normal tissue. We selected 10 types of relatively common cancer and collected the expression data of SLC25 family.

#### 2.1.4. Collection of Genome-Wide Mutation Data in Pan-Cancer Cell Lines

The Cancer Cell Line Encyclopedia (CCLE) database (https://portals.broadinstitute.org/ccle) was utilized to evaluate the expression differences, mutation, and copy number variation frequency of SLC25 family in 431 cell lines of 6 cancer types [7].

### 2.2. Multidimensional Analysis for the Expression of SLC25 Gene Family in Pan-Cancer

#### 2.2.1. Differential Expression Analysis of SLC25 Family in Pan-Cancer

The mRNA expression data of SLC25 family genes in pan-cancer was processed with Deseq2 R package to identify differentially expressed genes. The criteria were set as corrected *P* < 0.05 and at least 4-fold expression change (|logFc| ≥ 2).

The protein expression levels of SLC25 family in pan-cancer were assessed by the percentage of patients with high and medium protein expression in cancer tissue from the Human Protein Atlas (https://www.proteinatlas.org/) and cancer immunohistochemistry map.

Differentially expressed genes in pan-cancer cell lines were identified based on CCLE. The Kruskal-Wallis rank test was employed to compare the expression of SLC25 family genes in different cancer cell lines.

#### 2.2.2. Signal Transduction Pathway Analysis of SLC25 Family Genes in Pan-Cancer

The association between SLC25 family expression and tumor-related pathways was evaluated by Gene Set Variation Analysis (GSVA), which was a nonparametric method to estimate gene enrichment alteration with expression array samples [8]. The Pearson correlation coefficient (PCC) between SLC25 family expression and pathway activity was calculated to assess the association of SLC25 family with the activation or inhibition of some certain pathways. Pathways with |PCC| > 0.3 and adjusted *P* < 0.05 were considered to be significantly associated with SLC25 genes.

#### 2.2.3. Association Analysis of SLC25 Family Expression with Immune Cell Infiltration in Pan-Cancer

In a tumor microenvironment, metabolic disorder is an important link involved in the occurrence and development of tumors. The metabolic reprogramming of tumor microenvironment not only is the result of self-adaptation but also affects the surrounding immune cells to undergo metabolic reprogramming and lead to phenotypic changes [9]. Therefore, we further analyzed the relationship between SLC25 family and immune cell infiltration. The association between SLC25 family and immune cell infiltration was analyzed by calculating the Spearman correlation coefficient (SCC). Records with |SCC| > 0.3 and adjusted *P* <0.05 were determined to have statistical significance.

#### 2.2.4. Association Analysis of SLC25 Family Expression with Tumor Immune Microenvironment (TME) in Pan-Cancer

The stromal, immune, and ESTIMATE scores of each patient in each tumor were calculated based on gene expression of SLC25s using the R software package ESTIMATE. A higher immune score or stromal score means a higher ratio of the corresponding component in the TME. The estimate score is the sum of the two and represents the combined ratio of the two components in the TME, which can be used to predict tumor purity [10]. We used Spearman's correlation analysis between SLC25s expression and the ESTIMATE immune/stromal/estimate scores. A difference of *P* < 0.05 was considered significant.

#### 2.2.5. Association Analysis of SLC25 Family Expression with Immunotherapy Response in Pan-Cancer

We evaluated the predictive value of SLC25s in immunotherapeutic clinical benefits. Correlation of SLC25s with immune check point genes, such as PD-1, PD-L1, and CTLA4, was analyzed by TIMER2.0 database (http://timer.comp-genomics.org/). A difference of *P* < 0.05 was considered significant.

#### 2.2.6. Association Analysis of SLC25 Family Expression with Clinical Prognosis in Pan-Cancer

To investigate the association between SLC25 family genes and the survival of cancer patients, all cases were divided into two groups according to the median expression of SLC25 family. The intergroup survival rates were compared by a log-rank test, and *P* < 0.05 was regarded as statistically significant.

### 2.3. Analysis for the Mutation and Copy Number Variation of SLC25 Family in Pan-Cancer

#### 2.3.1. Mutation Analysis of SLC25 Family in Pan-Cancer

Based on the mutation data obtained from TCGA and CCLE, the percentage of gene mutation was determined as the mutation frequency of SLC25 family in cancer tissue and cell lines.

#### 2.3.2. Copy Number Variation Analysis of SLC25 Family in Pan-Cancer

The copy number variation of SLC25s in different cancer tissue and cell lines was identified based on the CNV data from TCGA and CCLE. Then, the CNV frequency in each tumor tissue and cell line was estimated with the ratio of CNV amplification and deletion.

### 2.4. Correlation Analysis of SLC25 Family Gene Mutation and Copy Number Variation with Their Expression in Pan-Cancer

The correlation of SLC25 family gene mutation and copy number variation with their expression was calculated with the Mann–Whitney *U* test by R software.

### 2.5. Validation by Quantitative PCR (qPCR) In Vivo

We collected clinical specimens from the First Hospital of China Medical University to validate the expression differences of SLC25 family genes in pan-cancer at mRNA level; differentially expressed SLC25 genes were validated in several cancers by real-time PCR. This study was reviewed and approved by the ethics committee of the First Hospital of China Medical University. In total, 23 pairs of gastric cancer and adjacent tissues, as well as 30 pairs of colorectal cancer and adjacent tissues, were included to detect the relative mRNA levels of SLC25s. Total RNA was extracted from samples using TRIzol. Relative quantification for SLC25A4, SLC25A7, and SLC25A23 in gastric cancer and colon cancer tissue was performed with SYBR Green kit (Takara, Japan) and real-time PCR system. The information of primer sequences is listed in Table S3. qPCR results were standardized with the 2^-*ΔΔ*CT^ method quantified by *β*-actin. And the specificity of PCR products was confirmed by melting curve analysis. The expression of SLC25 genes in tumor tissue was evaluated with a rank sum test in SPSS software, and *P* < 0.05 was considered statistically significant.

### 2.6. Validation by Immunohistochemistry (IHC) In Vivo

To further validate the predicted results of bioinformatics analysis, we validated the tissue protein levels of SLC25A4, SLC25A7, and SLC25A23 in gastric and colon cancers by IHC. Gastric cancer and colon cancer tissues were fixed in 4% paraformaldehyde, subsequently embedded in paraffin, and sectioned at 4 *μ*m. The expressions of SLC25A4 (rabbit anti-SLC25A4, 861244, Zenbio, Inc., Chengdu, China) in gastric cancer, UCP1 (rabbit anti-UCP1, 23673-1-AP, Proteintech Group, Inc., Wuhan, China) in gastric cancer and colon cancer, and SLC25A23 (rabbit anti-SLC25A23, 20168-1-AP, Proteintech Group, Inc., Wuhan, China) in colon cancer were analyzed by immunohistochemistry as per the manufacturer's instructions.

Percentage of stained cells (*S*) and immunostaining intensity (*I*) were assessed by two independent pathologists. Percentage scores ranged from 0 to 100%, and intensity scores ranged from 0 to 3 (0, no staining; 1, weak; 2, moderate; 3, strong). All fields of view were observed under a microscope. Different pathological changes were differentiated and scored by two experienced and highly qualified pathologists. The final IS score was obtained by multiplying the percentage (*S*) of stained cells by the intensity (*I*) (from 0 to 3). The data were analyzed using the SPSS statistical software 13.0 (SPSS Inc., Chicago, IL), and *P* < 0.05 was considered statistically significant.

## 3. Results

### 3.1. Expression Profiles of SLC25 Family in Pan-Cancer

#### 3.1.1. Expression of SLC25 Family at Tissue mRNA Level

**Figure 2:**
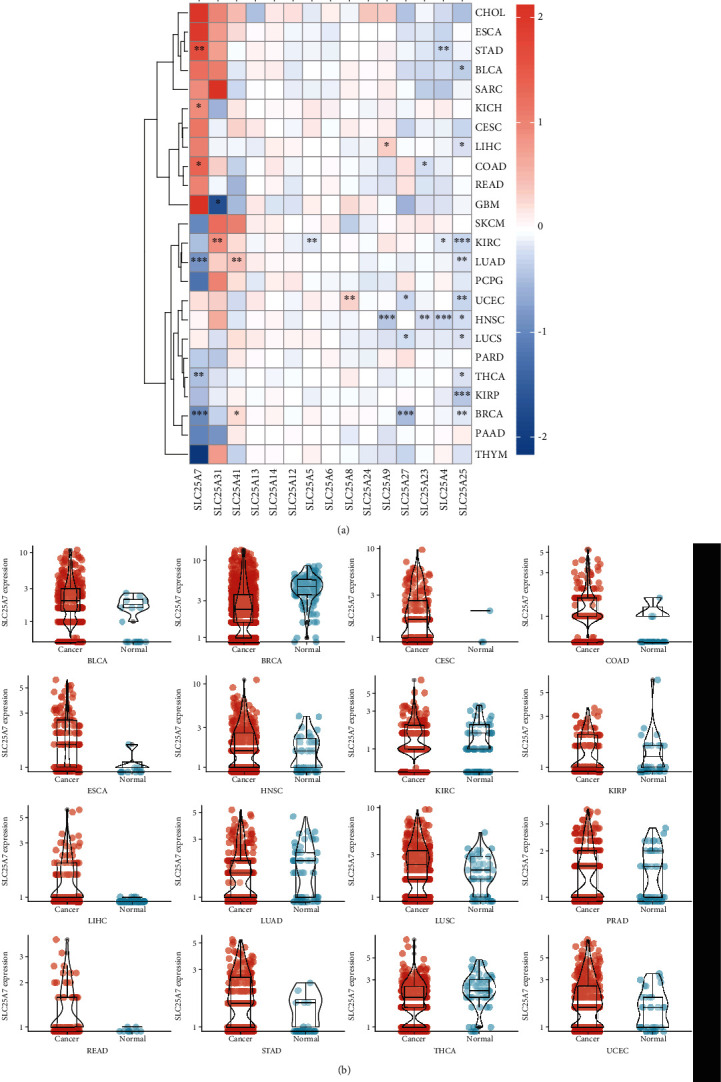
Expression of SLC25 family in different tumors at mRNA level. (a) The expression levels of SLC25 family in 33 cancer types. (b) The expression level of SLC25A7 in each cancer.

All 53 genes of SLC25 family were retrieved from published reviews for the detection of expression level. Due to the unknown function of some genes, we selected 15 genes of SLC25s with accurate function for the study. Foremost, the expression differences of SLC25s in various tumor tissue were analyzed based on TCGA data (Figure 2). The results showed that the SLC25 family was differentially expressed in 33 types of cancer. In stomach adenocarcinoma, SLC25A7 had increased expression while SLC25A4 was decreased. Only SLC25A25 was lowly expressed in bladder urothelial carcinoma. SLC25A7 was highly expressed in kidney chromophobe. SLC25A24 was upregulated in hepatocellular carcinoma while SLC25A25 was downregulated. In colon cancer, SLC25A7 expression was elevated while SLC25A23 was reduced. SLC25A31 was downregulated in glioblastoma multiform. In kidney renal clear cell carcinoma, all the expression of SLC25A31, SLC25A5, SLC25A4, and SLC25A25 was significantly decreased. In lung adenocarcinoma, SLC25A7 and SLC25A25 had decreased expression while SLC25A41 was elevated. SLC25A7 and SLC25A25 were reduced in uterine Corpus Endometrial Carcinoma. In head and neck squamous cell carcinoma, SLC25A9, SLC25A23, SLC25A4, and SLC25A5 all showed decreased expression. In lung squamous cell carcinoma, SLC25A27 and SLC25A25 were reduced. In thyroid cancer, significant downregulation was observed in SLC25A7 and SLC25A25. Besides, SLC25A25 was reduced in not only kidney renal papillary cell carcinoma but also breast invasive carcinoma. Moreover, SLC25A7 and SLC25A27 had decreased expression in breast invasive cancer while SLC25A41 was increased. Additionally, SLC25A25 was significantly downregulated in 10 of 33 types of cancer tissue (Figure 2(a)). The expression of SLC25A7 in each cancer was shown in Figure 2(b). Differential expression of other genes of the SLC25 family has been uploaded in Supplementary Materials (Figure S1).

#### 3.1.2. Expression of SLC25 Family at Tissue Protein Level

**Figure 3:**
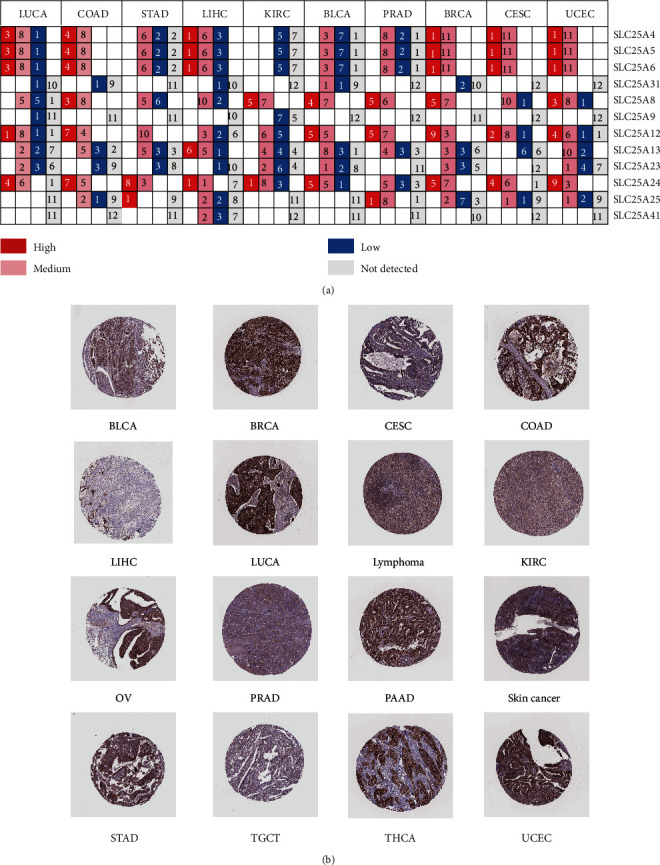
Expression of SLC25 family at protein level. (a) The expression levels of SLC25 proteins in 10 common cancer types. (b) The immunohistochemical map of SLC25A24 protein expression in different cancer tissue.

According to the immunohistochemical data from Human Protein Atlas, the SLC25 family had differential protein expression in different cancer tissue. The protein expression of SLC25A4, SLC25A5, and SLC25A6 was consistent in top 10 common cancer types. They were highly expressed in lung squamous cell carcinoma, colon adenocarcinoma, liver hepatocellular carcinoma, breast invasive carcinoma, cervical squamous cell carcinoma, endocervical adenocarcinoma, and uterine corpus endometrial carcinoma. SLC25A8 and SLC25A12 also had high expression levels in some cancer such as colon adenocarcinoma, bladder urothelial carcinoma, prostate adenocarcinoma, breast invasive cancer, and uterine corpus endometrial carcinoma. On the contrary, SLC25A23 and SLC25A13 had medium-to-low protein expression levels in some cancer. The expression of SLC25A9 and SLC25A31 was low or undetected. SLC25A24 was highly expressed in the 10 common cancer types (Figure 3(a)). The immunohistochemical data of SLC25A24 in different cancer tissue from the Human Protein Atlas showed that SLC25A24 had higher expression in 16 types of cancer including breast invasive cancer, colon adenocarcinoma, lung squamous cell carcinoma, prostate adenocarcinoma, stomach adenocarcinoma, thyroid carcinoma, and uterine corpus endometrial carcinoma (Figure 3(b)).

#### 3.1.3. Expression of SLC25 Family in Cell Lines

**Figure 4:**
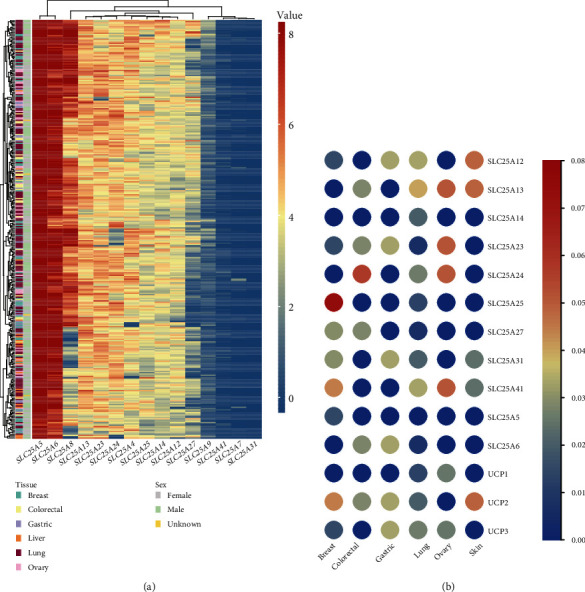
Expression of SLC25 family in cell lines. (a) The expression levels of SLC25 family in each cell line from CCLE. (b) The expression level of SLC25A5 gene in different cell lines from CCLE.

The analysis based on CCLE data suggested that some members of SLC25 family were highly expressed in breast, colorectal, stomach, liver, lung, and ovarian cancer cells, including SLC25A5, SLC25A6, and SLC25A8 (Figure 4(a)). SLC25A5 had high expression level in both male and female cases of cancer cells mentioned above. SLC25A6 was also increased in most cancer patients. SLC25A23 was mainly expressed in lung cancer and ovarian cancer cells to some extent. The expression level of SLC25A5 in different tumor cell lines from CCLE is shown in Figure 4(b).

### 3.2. Association of SLC25 Family Genes with Signaling Pathways in Pan-Cancer

**Figure 5:**
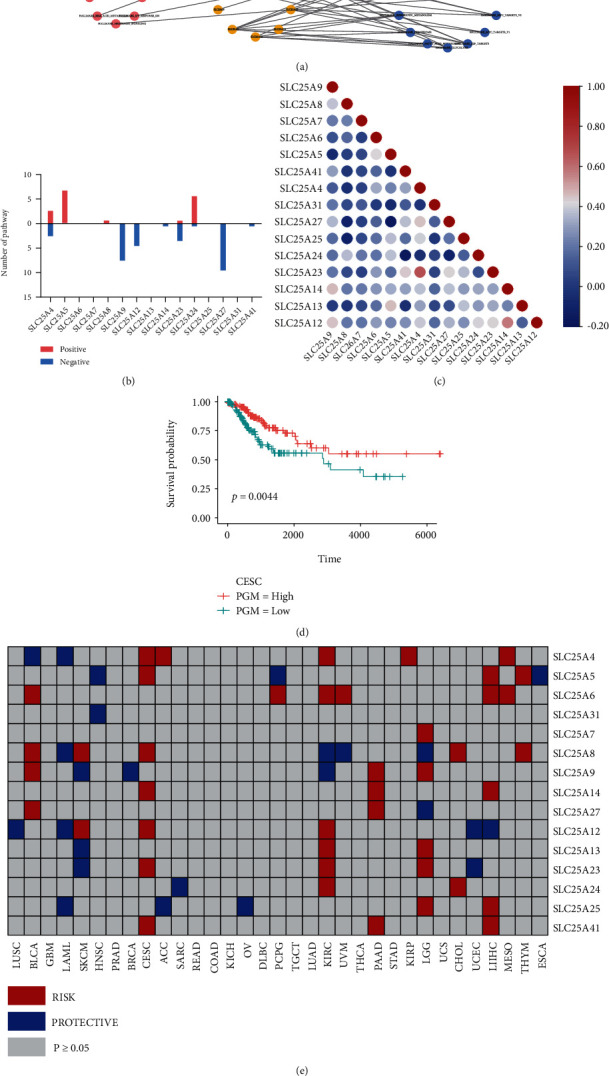
(a) The correlation between SLC25 family and tumor-related pathways. Yellow dots represent genes, red dots represent positively correlated pathways, and blue dots represent negatively correlated pathways. (b) The number of tumor-related pathways in SLC25 family. (c) The correlation between SLC25 family genes. Association of SLC25 family expression with prognosis. (d) The Kaplan-Meier survival curves of cervical squamous cell carcinoma and endocervical adenocarcinoma grouped by the overall expression pattern of SLC25A8. (e) The association between the expression of different genes in SLC25 family and prognosis.

Next, we analyzed and visualized the association of SLC25 family with signaling pathways in pan-cancer (*P* < 0.05, |*R*| > 0.3), and the correlation coefficient was calculated to explore the potential mechanism of SLC25 family in tumorigenesis (Figure 5(a)). The SLC25 family was shown to be associated with the activation or inhibition of various tumor-related pathways. SLC25A5 and SLC25A9 were more likely to be involved in carcinogenic pathways, mainly including PI3K_AKT_MTOR pathway, MYC_TARGETS_V1 pathway, MYC_TARGETS_V2 pathway, and MTORC1 pathway. Moreover, SLC25A23 and SLC25A4 were also related to these pathways. The number of oncogenic pathways associated with each gene of SLC25 and their influence on pathway activity are presented in Figure 5(b).

Furthermore, it was found that multiple genes of SLC25 could be involved in the same pathway with consistent correlation. For example, both SLC25A4 and SLC25A23 were negatively correlated with the E2F-TARGET and MYC-TARGET-V2 pathways. Besides, SLC25A12 and SLC25A14 were negatively correlated with the XENOBIOTIC-METABOLISM pathway. We speculated that the two genes involved in the same pathway might have synergistic effects or exert upstream and downstream regulatory roles in each other. Therefore, the correlation between these genes was further excavated suggesting that SLC25A4/SLC25A23 and SLC25A14/SLC25A12 might synergistically function in the E2F-TARGET, MYC-TARGET-V2, and XENOBIOTIC-METABOLISM pathways, respectively (Figure 5(c)).

### 3.3. Association of SLC25 Family Expression with Prognosis in Pan-Cancer

The prognostic significance of SLC25 family was evaluated by Cox regression analysis. The effects varied with SLC25 family genes and cancer types (Figure 5(e)). The SLC25 family was found to be significantly associated with the overall survival rate in at least one of 33 cancer types. Some genes of SLC25 were associated with poor prognosis of patients in cervical squamous cell carcinoma and endocervical adenocarcinoma, including SLC25A4, SLC25A5, SLC25A8, SLC25A14, SLC25A12, SLC25A23, and SLC25A41. In contrast, the expression of SLC25A4, SLC25A8, SLC25A12, and SLC25A25 was associated with better prognosis of patients in acute myeloid leukemia. Some other SLC25s made contrary effects on the prognosis of different cancers. For example, the SLC25A8 gene could lead to poor prognosis of patients in bladder urothelial carcinoma, skin cutaneous melanoma, cervical squamous cell carcinoma, endocervical adenocarcinoma, cholangiocarcinoma, and thymic carcinoma. However, it might prolong the survival in acute myeloid leukemia, kidney renal clear cell carcinoma, uveal melanoma, and brain low-grade glioma. As shown in Figure 5(d), SLC25A8 had significantly increased risk effects on the prognosis of cervical squamous cell carcinoma and adenocarcinoma.

### 3.4. The Role of SLC25s in the Tumor Microenvironment and Immunotherapy Response of Pan-Cancer

**Figure 6:**
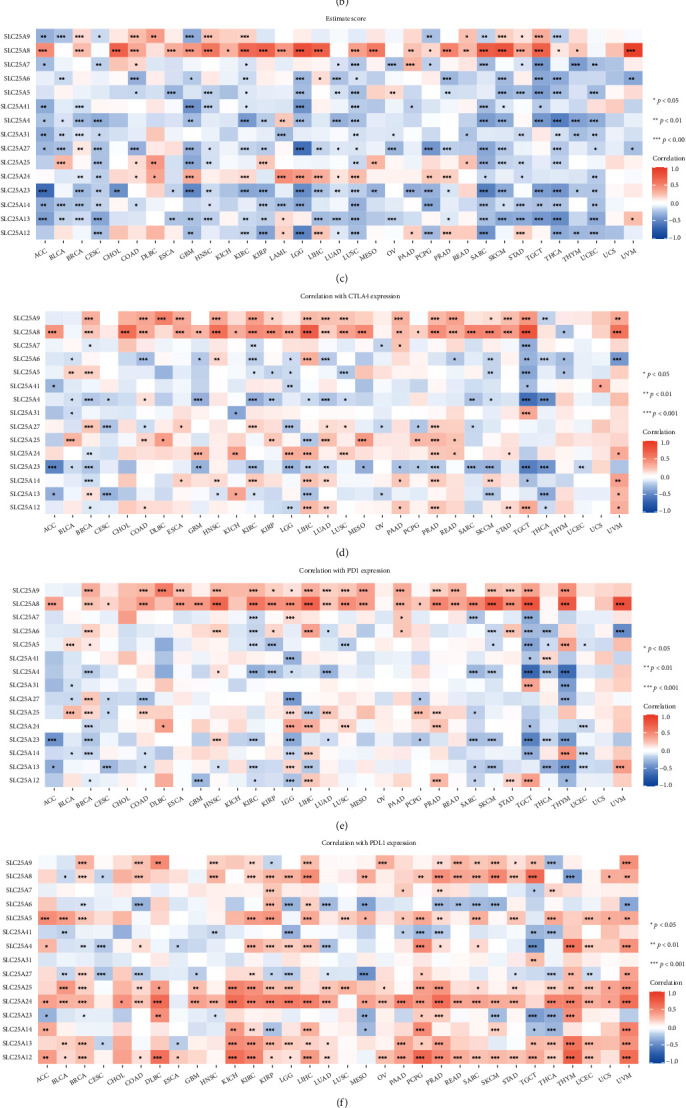
Association of SLC25 family with tumor immune cell infiltration, tumor microenvironment and immune check point inhibitor. (a–c) Heatmaps showing correlations of SLC25s with stromal score/immune score/estimate score. (d–f) The expression correlation between SLC25s and CTLA4/PD-1/PD-L1 in 33 cancer types. Only cells with *P* < 0.05 are shown in color. ^∗^*P* < 0.05,  ^∗∗^*P* < 0.01, and^∗∗∗^*P* < 0.001. (g) The maximal correlation between SLC25s and immune cell infiltration in each cancer tissue. (h) The correlation between SLC25 family and immune cell infiltration in all cancer tissue.

The ESTIMATE algorithm was used to analyze the relationship between SLC25 expression and tumor microenvironment in 33 cancer types. According to the results, majority of SLC25s expression were negatively correlated with stromal (Figure 6(a)), immune (Figure 6(b)), and estimate (Figure 6(c)) scores in most cancer types, while SLC25A8 expression was positively correlated with the majority cancers.

We further performed the correlation between the expression of SLC25s and immune check point gene expression to evaluate the potential immunotherapeutic response. Results showed that SLC25A8 was significantly positively associated with PD-1/PD-L1/CTLA4 expression in diverse cancer types, such as BRCA, COAD, HNSC, KIRC, KIRP, LGG, LIHC, TGCT, STAD, and UVM **(**Figures 6(d)–6(f)**)**.

### 3.5. Association of SLC25 Family with Tumor Immune Cell Infiltration

It is an accepted view that the metabolic reprogramming of cancer cells affects the activation or suppression of immune cells in tumor microenvironment. Immune cells also act on aerobic phosphorylation due to cancer cell stimulation and increase mitochondrial biogenesis [11, 12]. Then, the association of SLC25 family with immune cell infiltration in different cancer was investigated. The maximal correlation between those genes and each type of immune cell infiltration is shown in Figure 6(g). SLC25A6 was most correlated with macrophage M1 in uveal melanoma. Generally, the SLC25 family had strong correlation with the following immune cells including macrophages M2, T cell CD8, T cell CD4 memory activated, and T cell CD4 memory resting (Figure 6(h)).

### 3.6. Genetic Variation of SLC25 Family in Pan-Cancer

**Figure 7:**
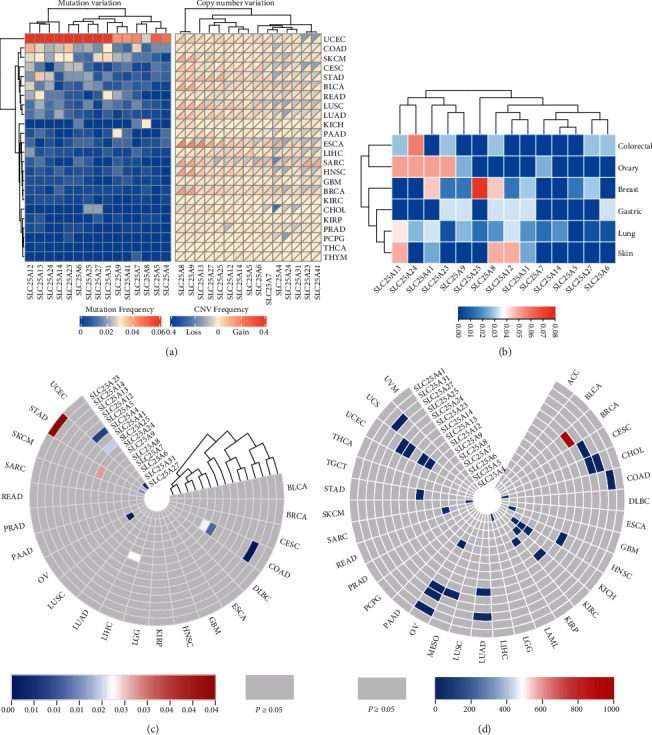
Genetic variation and effects of the mutation in SLC25 family on expression and cancer prognosis. (a) The mutation frequency and the copy number variation of SLC25s in different cancer tissue. (b) The mutation status of SLC25s in each cell line. (c) The heat map for the association between SLC25 family mutation and expression. (d) The heat map for the association between SLC25 family mutation and cancer prognosis.

Next, the mutation frequency of SLC25 family was investigated based on TCGA data. Almost all SLC25s had high mutation frequency in uterine corpus endometrial carcinoma, including SLC25A12, SLC25A13, SLC25A14, SLC25A23, SLC25A24, and SLC25A25. However, several genes such as SLC25A4 and SLC25A5 showed low mutation frequency in most cancer with an overall average mutation rate of 0.100189 (Figure 7(a)). Regarding the copy number variation (CNV) of SLC25 family, SLC25A8 demonstrated extensive CNV in different cancer types (Figure 7(a)). SLC25A4 and SLC25A24 had more copy number deletion in cholangiocarcinoma, sarcoma, and lung squamous cell carcinoma. In addition, the mutation of SLC25s was detected in different cancer cell lines based on CCLE data (Figure 7(b)). It was found that the mutation rate of SLC25A25 was the highest in breast cancer cell lines and SLC25A24 had a higher mutation level in colorectal cancer cell lines. Relatively high mutation rates were also observed in ovarian cancer cell lines for SLC25A13, SLC25A24, SLC25A41, and SLC25A23 and in skin cancer cell lines for SLC25A13, SLC25A8, and SLC25A12.

### 3.7. Effects of the Mutation in SLC25 Family on Expression and Cancer Prognosis

Then, we explored whether the mutation in the SLC25 family could influence their expression and cancer prognosis (Figure 7(c)). The mutation of SLC25A13 in colon cancer was suggested to have the most significant effect on its expression. In uterine corpus endometrial carcinoma, the mutation of SLC25A4, SLC25A25, and SLC25A27 had certain impacts on their expression. SLC25A23 mutation was significantly correlated with its expression in gastric cancer. In skin melanoma, the mutation of SLC25A24 and SCL25A27 could affect their expression. SLC25A6 mutation made significant effect on its expression in ovarian serous cystadenocarcinoma. SLC25A24 mutation could alter its expression in skin melanoma, hepatocellular carcinoma, and colon cancer. In colon cancer, both the expression of SLC25A13 and SLC25A9 could be affected by their mutation. All above-mentioned results reached statistical significance (*P* < 0.05).

Furthermore, association analysis was performed for the mutation of SLC25 family and cancer prognosis by calculating the hazard ratio (Figure 7(d)). SLC25A24 mutation showed the highest HR in breast invasive cancer among all SLC25 genes, and it also showed certain risk effects in mesothelioma, ovarian serous cystadenocarcinoma, and thyroid carcinoma. SLC25A25 mutation was associated with poor prognosis in cervical squamous cell carcinoma and adenocarcinoma, cholangiocarcinoma, and ovarian serous cystadenocarcinoma. Besides, the mutation of many SLC25s made high-risk effects on the prognosis of ovarian serous cystadenocarcinoma such as SLC25A8, SLC25A24, SLC25A25, and SLC25A31.

### 3.8. Effects of the Copy Number Variation in SLC25 Family on Expression

Other than gene mutation, we also studied the influence of copy number variation in the SLC25 family on gene expression in cancer. All the CNV of SLC25s were shown to affect their expression to some degrees. Among them, the CNV of SLC25A4 had statistical significance in most cancer, which was positively correlated with SLC25A4 expression (Table 1).

### 3.9. Validation of SLC25 Family Expression In Vivo by qPCR

**Figure 8:**
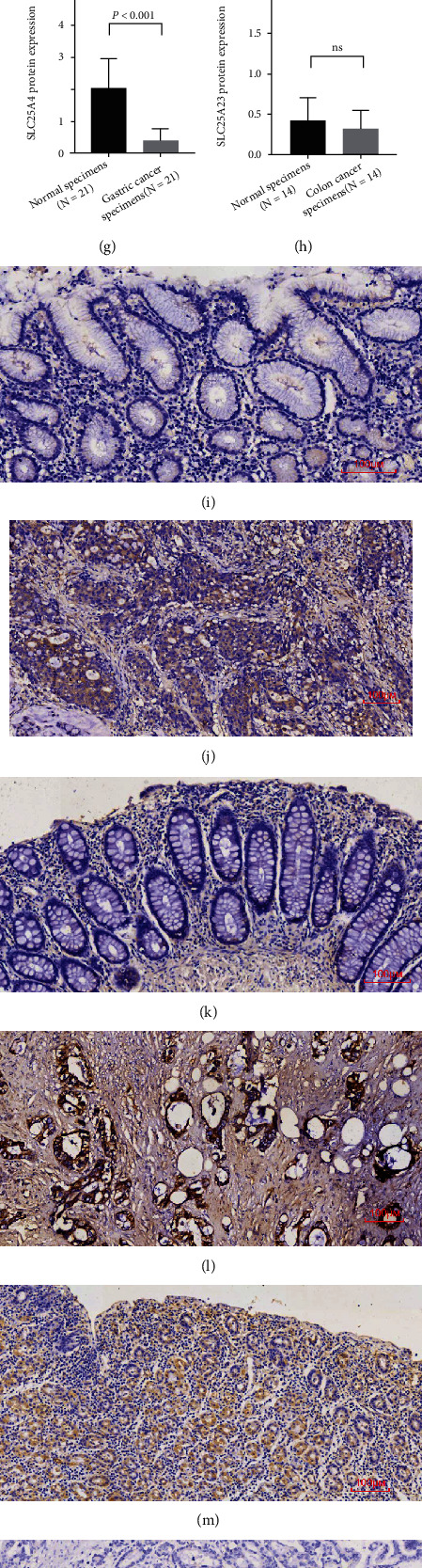
(a) The mRNA expression of SLC25A7 in gastric cancer specimens was increased; *N* = 23 per group (*P* = 0.023). (b) The mRNA expression SLC25A7 in colon cancer specimens was increased without statistical significance; *N* = 8 per group (ns). (c)The mRNA expression of SLC25A4 in gastric cancer specimens was decreased; *N* = 23 per group (*P* < 0.001). (d) The mRNA expression of SLC25A23 in colon cancer specimens was decreased; *N* = 29 per group (*P* < 0.001). (e) The protein expression of SLC25A7 in gastric cancer specimens was increased; *N* = 18 per group (*P* = 0.043). (f) The protein expression of SLC25A7 in colon cancer specimens was increased; *N* = 19 per group (*P* < 0.001). (g) The protein expression of SLC25A4 in gastric cancer specimens was decreased; *N* = 21 per group (*P* < 0.001). (h) The protein expression of SLC25A23 in COAD was decreased that did not reach the statistical significance; *N* = 14 per group (ns). (i) Representative immunostaining picture of SLC25A7 expression in gastric normal specimens. (j) Representative immunostaining picture of SLC25A7 expression in gastric cancer specimens. (k) Representative immunostaining picture of SLC25A7 expression in colon normal specimens. (l) Representative immunostaining picture of SLC25A7 expression in colon cancer specimens. (m) Representative immunostaining picture of SLC25A4 expression in gastric normal specimens. (n) Representative immunostaining picture of SLC25A4 expression in gastric cancer specimens. (o) Representative immunostaining picture of SLC25A23 expression in colon normal specimens. (p) Representative immunostaining picture of SLC25A23 expression in colon cancer specimens.

To validate the analytic results mentioned above, several differentially expressed genes were selected for qPCR validation, including SLC25A4 in gastric cancer, SLC25A23 in colon cancer, and SLC25A7 in gastric and colon cancer. It was found that SLC25A7 was significantly upregulated in gastric cancer (*P* = 0.023) (Figure 8(a)). The expression of SLC25A4 was decreased in STAD (*P* < 0.001) (Figure 8(c)). SLC25A23 had significantly low expression in colon cancer (*P* < 0.001) (Figure 8(d)), which was consistent with our bioinformatics prediction, while the alteration of SLC25A7 expression in COAD did not reach statistical significance (Figure 8(b)). However, the association of SLC25A4, SLC25A7, and SLC25A23 expression with clinicopathological parameters is not significant and detailed in Supplementary Table S4–5. And the original data for the association between the expression of SLC25A4, SLC25A7, and SLC25A23 and the clinicopathological parameters of gastric and colon cancer specimens are detailed in Table S6–9.

### 3.10. Validation of SLC25 Family Expression In Vivo by IHC

To verify the positive results in result 3.8, we further detected the expression of SLC25A7 in gastric and intestinal cancer, SLC25A4 in gastric cancer, and SLC25A23 in intestinal cancer by immunohistochemistry. By immunohistochemical staining, we observed that SLC25A4, SLC25A7, and SLC25A23 were mainly distributed in the cytoplasm. Our results showed that SLC25A7 was highly expressed in STAD and COAD, with a significant statistical difference between normal tissues (*P* = 0.043, *P* < 0.001) (Figures 8(e) and 8(f)). SLC25A4 expression was low in STAD and was statistically significantly different from normal tissue (*P* < 0.001) (Figure 8(g)). SLC25A23 expression was low in COAD and not statistically different from normal tissue, possibly related to the small number of samples (Figure 8(h)). In addition, the immunostaining of SLC25A4, SLC25A7, and SLC25A23 expression in gastric and colon cancer and the corresponding paracancerous tissues of the same patient were shown (Figures 8(i)–8(p)).

## 4. Discussion

In the present study, we explored the roles of SLC25 family in the genesis and development of cancer. According to current publications, a total of 15 SLC25 family genes were screened out. Multidimensional analyses were systematically performed based on the transcriptome and genome data of the SLC25 family from a variety of online databases for the association among their immune cell infiltration, tumor microenvironment, immunotherapeutic response, expression, mutation and copy number variation, cancer prognosis, and signaling pathways. Validation by qPCR and IHC was further conducted for the expression status of partial SLC25 family members in some tumor tissues.

Our study demonstrated that the SLC25 family had differential expression in both tissue mRNA and protein as well as cell lines. For detailed results at tissue mRNA level, SLC25A4 was lowly expressed in stomach adenocarcinoma and colon adenocarcinoma. SLC25A7 was highly expressed in stomach adenocarcinoma and colon adenocarcinoma. SLC25A23 had low expression in colon cancer. Therefore, SLC24A4, SLC25A7, and SLC25A23 were selected for subsequent validation of in situ expression in isolated fresh human cancer tissue by qPCR and IHC. At the mRNA level of specimens, we found that SLC25A4 and SLC25A23 were, respectively, decreased in gastric cancer and colon cancer, while SLC25A7 was increased in gastric cancer, which was consistent with our bioinformatics prediction. Though SLC25A7 was increased in colon cancer, the result did not reach statistical significance. Further, we verified the expression of the above molecules from the tissue protein level by IHC. The results showed that SLC25A7 was upregulated in gastric cancer and colon cancer specimens. SLC25A4 was downregulated in gastric cancer specimens, while SLC25A23 was downregulated in colon cancer specimens without statistical significance. This may be related to the few samples or the existence of posttranscriptional regulation. It has been shown that the ANT1 protein encoded by SLC25A4 was also lowly expressed in rhabdomyosarcoma [13]. Combining with this, ANT1 transports ADP to mitochondrial matrix and synthesizes ATP there, then exports it to the cytoplasm to provide fuel for the metabolic energy process [14]. SLC25A4 is located in mitochondria and involved in metabolic process by regulating ATP/ADP. Thus, the decreased expression of SLC25A4 might lead to aberrant metabolism, participating in tumor progression. As a well-known uncoupling protein, UCP1 encoded by SLC25A7 can gently uncouple oxidative phosphorylation or reduce the generation of ROS from the perspective of lipid metabolism with protecting cells from oxidative stress [15, 16]. This function enables UCP1 to produce a large amount of heat in the brown fat of newborns, while its effect in cancer has been rarely studied. Similar findings could be observed in previous research for the expression of SLC25A7 in tumor. Alexandra et al. reported that SLC25A7 was highly expressed in squamous cell carcinoma of non-small cell lung cancer and closely related to glycolysis [17]. Cancer cells can reduce the influx of calcium to reduce apoptosis [18]. SLC25A23 acts as a Ca2+-sensitive mitochondrial carrier; it can decrease the expression of SLC25A23 by reducing Ca2+, which in turn affects the production of ROS at different locations. SLC25A23 is a mitochondrial carrier with the ability to effectively transport ATP, ADP, and AMP except for Ca2+. The deletion of SLC25A23 reduces oxidative phosphorylation [19], and SLC25A23 can maintain mitochondrial ATP levels [20]. The study would provide theoretical basis for their application as potential targets of cancer therapy including SLC25A4, SLC25A7, and SLC25A23.

The association analysis of SLC25 expression with oncogenic pathways revealed 35 pathways associated with SLC25s such as proliferation-related pathways, Kas and MYC, and metabolic pathways. The expression of SLC25 genes varied with each pathway, suggesting that different SLC25 genes might exert diverse roles in tumor. Among them, SLC25A5 was more likely to be involved in carcinogenic pathways. It has been reported that the SLC25A5 gene is upregulated in most tumor [21–24], mainly encoding the ANT2 protein specifically expressed in proliferative tissue [25]. Several pathways shown to be positively associated with SLC25A5 were common oncogenic pathways regulating proliferation, including PI3K_AKT_MTOR, MYC_TARGETS_V2, and E2F_TARGETS [26–29]. ANT inhibitors have been considered potential therapeutic targets for cancer [30]. It is worth noting that in the oncogenic pathways of the SLC25 family, we found that SLC25A4 is positively correlated with the bile acid metabolism pathway. A new study found that in the type III metabolic subtypes with poor clinical features, bile acids were abnormally activated and the disorder of bile acid metabolism promoted the metastasis of lung adenocarcinoma (LUAD) through metabolomics cluster analysis of patients with invasive lung adenocarcinoma. [31]. Therefore, signaling pathways related to bile acid metabolism may be a potential therapeutic target for invasive lung adenocarcinoma, as shown in our results for SLC25A4. Except for the pathways that are positively related to SLC25 family genes, our results showed SLC25A9 and SLC25A12 were negatively related to fatty acid metabolism. The latest research revealed that LPL/FABP4/CPT1, a fatty acid metabolism reprogramming axis, was significantly upregulated in nonalcoholic steatohepatitis (NASH) and cooperated with a large number of oncogenic signals to drive NASH to directly progress to hepatocellular carcinoma. For this reason, SLC25A9 and SLC25A12 may become therapeutic targets for hepatocellular carcinoma through fatty acid metabolism [32]. Combining previous researches and our results, we speculated SLC25A4, SLC25A5, SLC25A9, and SLC2512 might participate in metabolic reprogramming and cancer initiation and development via these oncogenic and metabolic pathways. To elucidate the association hidden in the SLC25 family genes differentially expressed in tumor could not only benefit the identification of novel molecules involved in the regulation of tumor progression and better insights of pathogenesis but also provide clues for candidate biomarkers used for cancer diagnosis, prognosis, and treatment.

A large number of studies have reported that in tumor microenvironment, metabolic disorders were a crucial link involved in the initiation and development of cancer [33]. As the close connection between the above SLC25 family genes and metabolic pathways, we analyzed the association between the SLC25 family and tumor microenvironment, immunotherapeutic response, and the immune cell infiltration. The SLC25 family was previously reported to be relevant with inflammation [34]. Yasukawa et al. found that SLC25A12 was associated with the innate immunity of body through mitochondrial metabolism [35]. Our results suggest that SLC25s may play a key role in the tumor microenvironment. In majority cancers, the expression of most SLC25s was negatively correlated with stromal/immune/estimate scores. With the deepening of the research on the tumor immune microenvironment, the ability to predict and guide the response to immunotherapy has great development and research potential [36]. At present, immune checkpoint inhibitors (ICIs) are the most widely used in cancer immunotherapy, and anti-CTLA4-PD-1 dual immunotherapy has successfully treated a group of tumors including melanoma [37], so we selected the common ICIs—PD-1, PD-L1, and CTLA4, to explore the role of SLC25s in cancer immunotherapy. The results showed SLC25s are often associated with the expression of PD-1/PD-L1/CTLA4, implying that SLC25s may have potential value in predicting immunotherapy response. Alves-Guerra et al. showed that SLC25A8 was rapidly activated and mobilized during the anesthesia of immune cells [38]. Hence, the SLC25 family (such as SLC25A16 [39], SLC25A12 [35], and SLC25A27 [40]) was related to immunity more or less. In our research, the association between SLC25s and immune cell infiltration was mainly manifested in macrophage M2. The role of macrophages in tumorigenesis and development has already been confirmed. They had a two-way interaction with cancer cells, especially M2 polarization. Cancer cells induced M2 polarity to promote a series of cancer deterioration [41]. Various metabolites in tumor microenvironment promoted the M2 polarization of macrophages to accelerate tumor progression [42]. The latest research showed that increased expression of heme oxygenase (HO-1) in tumor-associated macrophages (TAMs) could promote tumor metastasis through M2 polarization of macrophages [43]. TAMs might regulate the expression level of HO-1 by regulating SLC25A4 to promote the M2 polarization of macrophages and enhance the ability of tumor metastasis. These may provide corresponding thoughts for the diagnosis and treatment of cancer with respect to SLC25 family and tumor microenvironment.

Concerning the relationship of SLC25s expression with cancer prognosis, SLC25A8 was associated with poor prognosis in cervical squamous cell carcinoma, while better prognosis in membranous melanoma and brain low-grade glioma. A latest study by Wang et al. demonstrated that knocking down SLC25A8 in cervical cancer cell lines could delay or reduce the proliferation, migration, and invasion of cervical cancer cells via Ras/MAPK/ERK signaling pathways. Thus, it could be applied to predicting the prognosis of cervical cancer [44], which was consistent with our findings. Similarly, research for breast cancer indicated that SLC25A8 overexpression might directly affect the mitochondrial membrane potential of tumor cells, inhibit cell apoptosis, and promote tumor metastasis through the TGF-*β* pathway, resulting in the poor prognosis [45]. Kuai et al. also believed that SLC25A8 was highly expressed in human colon cancer tissue associated with the metastasis and poor prognosis of colon cancer [46]. Furthermore, we found that SLC25A8 made discrepant effects on the prognosis of different cancer, with the potential to be a versatile biomarker for cancer prognosis.

Currently, numerous studies have proved that the occurrence and progression of tumor were closely related to genetic variation including mutation and copy number variation. The mutation and copy number variation of SLC25 family were also investigated based on TCGA data. It was shown that almost all SLC25 genes had high mutation frequency in uterine corpus endometrial carcinoma, which was a well-accepted tumor with extensive gene mutation [47]. Notably, SLC25A4 had more deletion of copy number in cholangiocarcinoma, sarcoma and lung squamous cell carcinoma. For its reason, the expression of ANT1 protein encoded by SLC25A4 might reduce ATP transport and promote Rax release, easily leading to cell apoptosis and tumorigenesis [48–50]. Moreover, the mutation analysis of SLC25 family in tumor cell lines revealed that the mutation rate of SLC25A25 was the highest in breast cancer, suggesting that SLC25A25 was more apt to mutate in the environment of breast tissue. The SLC25A25 gene could control the dynamic balance of ATP, and the missing of SLC25A25 may reduce the metabolic efficiency in mice [51]. We also analyzed the influence of mutation and CNV in SLC25 genes on their expression and cancer prognosis. It has been reported that cells pretreated with ANT inhibitors can attenuate the release of Cytc connected with mitochondrial bioenergy. Therefore, ANT mutations are involved in many human diseases [52], which is consistent with our research. As for the impacts of mutation on cancer prognosis. Besides, SLC25A24 could protect tumor cells from death. Thus, it might promote tumor growth and metastasis causing poor prognosis when mutated [53]. The variation in SLC25 genes might play critical roles in the regulation of their expression and cancer prognosis. However, the specific mechanism needs to be further explored.

## 5. Conclusion

In summary, multidimensional analyses were systematically performed for the differential expression of SLC25 genes in 33 types of human cancer. Several genes were selected for the validation of expression in fresh cancer tissue by qPCR and IHC including SLC25A4, SLC25A7, and SLC25A23. The results showed that SLC25A4 and SLC25A23 were, respectively, decreased in gastric cancer and colon cancer, while SLC25A7 was increased in gastric cancer, which was consistent with our bioinformatics prediction. It may provide theoretical basis for their application as novel targets of cancer therapy. SLC25A4 and SLC25A5 might be involved in some carcinogenic pathways and metabolic pathways regulated by genetic variation, participating in cancer initiation and development. In addition, we also investigated the association of SLC25s with clinical prognosis and tumor microenvironment, immunotherapeutic response and immune infiltration. All these findings suggested that the SLC25 family participated in immune microenvironment and might be crucial to the occurrence, progression, and prognosis of tumor. They could be considered predictive biomarkers for early diagnosis and prognosis as well as potential targets for individualized treatment of cancer.

## Figures and Tables

**Table 1:** Association of the copy number variation in SLC25A4 gene with expression in pan-cancer.

Cancer Type	CNVCat	*n*	Summarise	*P*
ACC	DEL	9	11.176 (10.262-11.231)	0.228
	GAIN	3	12.671 (11.437-12.876)	
	No change	65	11.218 (10.523-11.758)	

BLCA	DEL	29	9.557 (9.101-9.994)	**0.004**
	GAIN	14	10.327 (9.858-11.015)	
	No change	365	10.192 (9.64-10.773)	

BRCA	DEL	68	10.019 (9.581-10.474)	**< 0.001**
	GAIN	49	11.231 (10.452-11.872)	
	No change	973	10.75 (10.176-11.244)	

CESC	DEL	23	10.18 (9.687-10.803)	**0.011**
	GAIN	5	11.746 (11.022-11.769)	
	No change	266	10.705 (10.049-11.359)	

CHOL	DEL	9	9.973 (9.822-10.372)	0.056
	No change	27	10.522 (10.076-11.277)	

COAD	DEL	40	9.618 (8.937-10.178)	**< 0.001**
	GAIN	2	10.604 (9.971-11.237)	
	No change	420	10.18 (9.592-10.718)	

DLBC	DEL	5	9.806 (9.058-10.067)	0.055
	GAIN	1	9.656 (9.656-9.656)	
	No change	42	10.609 (9.905-11.024)	

ESCA	DEL	14	9.951 (9.557-10.403)	**< 0.001**
	GAIN	13	11.345 (10.706-11.372)	
	No change	134	10.383 (9.884-10.824)	

GBM	DEL	8	11.743 (11.532-11.963)	0.117
	No change	156	11.982 (11.644-12.315)	

HNSC	DEL	48	9.446 (8.75-10.048)	**0.002**
	GAIN	10	10.423 (10.242-11.867)	
	No change	438	9.972 (9.189-10.592)	

KICH	DEL	2	13.39 (12.964-13.816)	0.556
	No change	63	13.946 (13.593-14.502)	

KIRC	DEL	11	10.913 (10.672-11.107)	**0.004**
	GAIN	1	12.21 (12.21-12.21)	
	No change	518	11.608 (11.072-12.051)	

KIRP	DEL	3	11.012 (10.809-11.651)	0.269
	GAIN	2	10.922 (10.708-11.135)	
	No change	282	11.736 (11.194-12.2)	

LGG	DEL	35	11.51 (11.286-12.085)	**< 0.001**
	GAIN	2	12.677 (12.492-12.861)	
	No change	491	12.347 (11.996-12.729)	

LIHC	DEL	35	10.465 (9.772-10.904)	**< 0.001**
	GAIN	9	11.628 (11.576-12.42)	
	No change	328	11.12 (10.557-11.564)	

LUAD	DEL	37	10.195 (9.633-10.788)	**0.002**
	GAIN	11	11.458 (10.399-11.682)	
	No change	476	10.753 (10.138-11.284)	

LUSC	DEL	54	10.167 (9.589-10.647)	**< 0.001**
	GAIN	20	11.387 (10.896-12.177)	
	No change	426	10.47 (10.043-10.985)	

MESO	DEL	4	9.928 (9.569-10.411)	0.166
	No change	82	10.792 (10.155-11.309)	

OV	DEL	67	10.831 (10.365-11.339)	**< 0.001**
	GAIN	31	11.933 (11.233-12.217)	
	No change	279	11.238 (10.754-11.682)	

PAAD	DEL	9	9.745 (9.426-10.207)	**0.015**
	No change	168	10.339 (9.954-10.726)	

PCPG	DEL	6	11.862 (11.713-11.971)	**0.016**
	GAIN	1	11.741 (11.741-11.741)	
	No change	161	12.479 (12.141-12.802)	

PRAD	DEL	15	11.313 (10.6-11.549)	**0.005**
	GAIN	7	11.825 (11.563-11.945)	
	No change	474	11.767 (11.443-12.109)	

READ	DEL	8	9.938 (8.942-10.536)	0.796
	GAIN	4	9.969 (9.6-10.479)	
	No change	153	10.04 (9.553-10.564)	

SARC	DEL	51	9.053 (8.112-10.17)	**< 0.001**
	GAIN	12	9.296 (8.636-10.487)	
	No change	199	10.105 (9.144-11.82)	

SKCM	DEL	26	11.108 (10.354-11.807)	**< 0.001**
	GAIN	16	12.591 (11.896-12.954)	
	No change	428	11.783 (11.008-12.553)	

STAD	DEL	44	10.278 (9.867-10.524)	**< 0.001**
	GAIN	9	11.171 (10.814-11.71)	
	No change	320	10.683 (10.087-11.241)	

TGCT	DEL	1	6.7 (6.7-6.7)	0.121
	GAIN	5	10.29 (9.896-10.485)	
	No change	150	9.4 (8.7-10.101)	

THCA	DEL	1	9.953 (9.953-9.953)	0.060
	GAIN	1	10.513 (10.513-10.513)	
	No change	505	11.79 (11.458-12.134)	

UCEC	DEL	24	9.61 (8.922-10.068)	**0.002**
	GAIN	10	11.218 (10.123-11.712)	
	No change	506	10.083 (9.553-10.675)	

UCS	DEL	3	10.721 (10.463-10.988)	0.352
	GAIN	3	11.58 (11.228-11.813)	
	No change	49	10.892 (10.485-11.326)	

UVM	GAIN	1	8.48 (8.48-8.48)	0.091
	No change	79	10.995 (10.14-11.687)	

Note: The results are in bold if *P* <0.05.

## Data Availability

The data that support the findings of this study are openly available in The Cancer Genome Atlas (TCGA) data portal (https://tcga-data.nci.nih.gov/tcga/), Human Protein Atlas (https://www.proteinatlas.org/), Gene Cards (https://www.genecards.org/), UCSC Xena (https://Xenabrowser.net/), and Cancer Cell Line Encyclopedia (CCLE) database (https://portals.broadinstitute.org/ccle). The rest of the data are available from the corresponding author on reasonable request.

## Abbreviations

SLC25: Solute carrier family 25
qPCR: Quantitative Real-time PCR
OMM: Outer mitochondrial membrane
IMM: Inner mitochondrial membrane
HGNC: HUGO Gene Nomenclature Committee
TCGA: The Cancer Genome Atlas
TPM: Transcripts Per Kilobase Million
CCLE: Cancer Cell Line Encyclopedia
GSVA: Gene Set Variation Analysis
PCC: Pearson correlation coefficient
SCC: Spearman correlation coefficient
CNV: Copy number variation.

## Supplementary Materials

Supplementary Materials Table S1: the genes of SLC25 family and its references. Table S2: the abbreviation of 33 cancer types. Table S3: the information of primer sequences. Table S4: the correlation of SLC25A4&SLC25A7 expression and clinical pathological parameters in gastric cancer. Table S5: the correlation of SLC25A23&SLC25A7 expression and clinical pathological parameters in colon cancer. Table S6: the original data for the association between the expression of SLC25A4 and the clinicopathological parameters of gastric cancer specimens. Table S7: the original data for the association between the expression of SLC25A7 and the clinicopathological parameters of gastric cancer specimens. Table S8: the original data for the association between the expression of SLC25A7 and the clinicopathological parameters of colon cancer specimens. Table S9: the original data for the association between the expression of SLC25A23 and the clinicopathological parameters of colon cancer specimens. Figure S1: the differential expression of other genes of SLC25 family. Figure S1 legend. The legend of Figure S1.
