# Supplementary material for: Comprehensive Analysis and Validation of Solute Carrier Family 25 (SLC25) and Its Correlation with Immune Infiltration in Pan-Cancer
Source: Biomed Res Int. 2022 Oct 8;2022:4009354. doi: 10.1155/2022/4009354 (PMC9569204; doi:10.1155/2022/4009354)
Supplement: Supplementary Materials — Table S1: the genes of SLC25 family and its references. Table S2: the abbreviation of 33 cancer types. Table S3: the information of primer sequences. Table S4: the correlation of SLC25A4&SLC25A7 expression and clinical pathological parameters in gastric cancer. Table S5: the correlation of SLC25A23&SLC25A7 expression and clinical pathological parameters in colon cancer. Table S6: the original data for the association between the expression of SLC25A4 and the clinicopathological parameters of gastric cancer specimens. Table S7: the original data for the association between the expression of SLC25A7 and the clinicopathological parameters of gastric cancer specimens. Table S8: the original data for the association between the expression of SLC25A7 and the clinicopathological parameters of colon cancer specimens. Table S9: the original data for the association between the expression of SLC25A23 and the clinicopathological parameters of colon cancer specimens. Figure S1: the differential expression of other genes of SLC25 family. Figure S1 legend. The legend of Figure S1. [file 4009354.f1.zip › Figure S1 legend (1).docx]

Figure S1. The differential expression of other genes of SLC25 family. SLC25A47 was significant down-regulated in KICH and CHOL. SLC25A52 was significant down-regulated in CHOL. SLC25A48 was up-regulated in LUAD, LUSC, UCEC, PRAD, THCA and down-regulated in KIRC, KIRP, LIHC, COAD, KICH and CHOL.
