# Supplementary material for: Comprehensive Analysis and Validation of Solute Carrier Family 25 (SLC25) and Its Correlation with Immune Infiltration in Pan-Cancer
Source: Biomed Res Int. 2022 Oct 8;2022:4009354. doi: 10.1155/2022/4009354 (PMC9569204; doi:10.1155/2022/4009354)
Supplement: Supplementary Materials — Table S1: the genes of SLC25 family and its references. Table S2: the abbreviation of 33 cancer types. Table S3: the information of primer sequences. Table S4: the correlation of SLC25A4&SLC25A7 expression and clinical pathological parameters in gastric cancer. Table S5: the correlation of SLC25A23&SLC25A7 expression and clinical pathological parameters in colon cancer. Table S6: the original data for the association between the expression of SLC25A4 and the clinicopathological parameters of gastric cancer specimens. Table S7: the original data for the association between the expression of SLC25A7 and the clinicopathological parameters of gastric cancer specimens. Table S8: the original data for the association between the expression of SLC25A7 and the clinicopathological parameters of colon cancer specimens. Table S9: the original data for the association between the expression of SLC25A23 and the clinicopathological parameters of colon cancer specimens. Figure S1: the differential expression of other genes of SLC25 family. Figure S1 legend. The legend of Figure S1. [file 4009354.f1.zip › Table S1 Genes of SLC25 family (1).docx]

**Table S2** genes of SLC25 family

| SLC25 family | abbreviate | Function | reference |
| --- | --- | --- | --- |
| SLC25A1 | CIC | citrate (tricarboxylate) carrier | [1] |
| SLC25A2 | ORC2 | ornithine carriers | [2] |
| SLC25A3 | PiC | Pi carrier | [3] |
| SLC25A4 | ANT1 | adenine nucleotide translocases | [4] |
| SLC25A5 | ANT2 | adenine nucleotide translocases | [4] |
| SLC25A6 | ANT3 | adenine nucleotide translocases | [4] |
| SLC25A7 | UCP1 | uncoupling proteins | [5] |
| SLC25A8 | UCP2 | uncoupling proteins | [5] |
| SLC25A9 | UCP3 | uncoupling proteins | [5] |
| SLC25A10 | DIC | dicarboxylate carrier | [6] |
| SLC25A11 | OGC | oxoglutarate carrier | [7] |
| SLC25A12 | AGC1 | Ca2+-sensitive mitochondrial carrier | [8] |
| SLC25A13 | AGC2 | Ca2+-sensitive mitochondrial carrier | [8] |
| SLC25A14 | UCP5 | uncoupling proteins | [5] |
| SLC25A15 | ORC1 | ornithine carriers | [2] |
| SLC25A16 | GDC | Grave’s disease carrier | [9] |
| SLC25A17 | ANC | the peroxisomal transporter | [10] |
| SLC25A18 | GC2 | glutamate carriers | [11] |
| SLC25A19 | DNC | deoxynucleotide carrier | [12] |
| SLC25A20 | CAC | carnitine/acylcarnitine carriers | [13] |
| SLC25A21 | ODC | oxoadipate carrier | [14] |
| SLC25A22 | GC1 | glutamate carriers | [11] |
| SLC25A23 | APC2 | Ca2+-sensitive mitochondrial carrier | [15] |
| SLC25A24 | APC1 | Ca2+-sensitive mitochondrial carrier | [15] |
| SLC25A25 | APC3 | Ca2+-sensitive mitochondrial carrier | [15] |
| SLC25A26 | SAMC | S-adenosylmethionine carrier | [16] |
| SLC25A27 | UCP4 | uncoupling proteins | [5] |
| SLC25A28 | MFRN2 | iron carriers | [17] |
| SLC25A29 | CACL | carnitine/acylcarnitine carriers | [18] |
| SLC25A30 |  |  |  |
| SLC25A31 | AAC4 | adenine nucleotide translocases | [4] |
| SLC25A32 | MFT | folate carrier | [19] |
| SLC25A33 | PNC1 | pyrimidine nucleotide carriers | [20] |
| SLC25A34 |  |  |  |
| SLC25A35 |  |  |  |
| SLC25A36 | PNC2 | pyrimidine nucleotide carriers | [20] |
| SLC25A37 | MFRN1 | iron carriers | [17] |
| SLC25A38 | GLYC |  | [21] |
| SLC25A39 |  |  |  |
| SLC25A40 |  |  |  |
| SLC25A41 | APC4 |  | [22] |
| SLC25A42 |  | CoA carrier | [23] |
| SLC25A43 |  |  |  |
| SLC25A44 |  |  |  |
| SLC25A45 |  |  |  |
| SLC25A46 |  |  |  |
| SLC25A47 |  |  |  |
| SLC25A48 |  |  |  |
| SLC25A49 | MTCH1 |  | [24] |
| SLC25A50 | MTCH2 |  | [25] |
| SLC25A51 | MCART1 | NAD(+) transporter | [26] |
| SLC25A52 | MCART2 | NAD(+) transporter |  |
| SLC25A53 | MCART6 | NAD(+) transporter |  |

1. Majd, H., et al., *Pathogenic mutations of the human mitochondrial citrate carrier SLC25A1 lead to impaired citrate export required for lipid, dolichol, ubiquinone and sterol synthesis.* Biochim Biophys Acta Bioenerg, 2018. **1859**(1): p. 1-7.

2. Palmieri, L., et al., *Identification of the yeast ARG-11 gene as a mitochondrial ornithine carrier involved in arginine biosynthesis.* FEBS Lett, 1997. **410**(2-3): p. 447-51.

3. Kunji, E.R. and A.J. Robinson, *Coupling of proton and substrate translocation in the transport cycle of mitochondrial carriers.* Curr Opin Struct Biol, 2010. **20**(4): p. 440-7.

4. Bamber, L., et al., *The yeast mitochondrial ADP/ATP carrier functions as a monomer in mitochondrial membranes.* Proc Natl Acad Sci U S A, 2007. **104**(26): p. 10830-4.

5. Aquila, H., T.A. Link, and M. Klingenberg, *The uncoupling protein from brown fat mitochondria is related to the mitochondrial ADP/ATP carrier. Analysis of sequence homologies and of folding of the protein in the membrane.* EMBO J, 1985. **4**(9): p. 2369-76.

6. Mizuarai, S., et al., *Identification of dicarboxylate carrier Slc25a10 as malate transporter in de novo fatty acid synthesis.* J Biol Chem, 2005. **280**(37): p. 32434-41.

7. Iacobazzi, V., et al., *Sequences of the human and bovine genes for the mitochondrial 2-oxoglutarate carrier.* DNA Seq, 1992. **3**(2): p. 79-88.

8. Palmieri, L., et al., *Citrin and aralar1 are Ca(2+)-stimulated aspartate/glutamate transporters in mitochondria.* EMBO J, 2001. **20**(18): p. 5060-9.

9. Zarrilli, R., et al., *Sequence and chromosomal assignment of a novel cDNA identified by immunoscreening of a thyroid expression library: similarity to a family of mitochondrial solute carrier proteins.* Mol Endocrinol, 1989. **3**(9): p. 1498-505.

10. Agrimi, G., et al., *The human gene SLC25A17 encodes a peroxisomal transporter of coenzyme A, FAD and NAD+.* Biochem J, 2012. **443**(1): p. 241-7.

11. Fiermonte, G., et al., *Identification of the mitochondrial glutamate transporter. Bacterial expression, reconstitution, functional characterization, and tissue distribution of two human isoforms.* J Biol Chem, 2002. **277**(22): p. 19289-94.

12. Lindhurst, M.J., et al., *Knockout of Slc25a19 causes mitochondrial thiamine pyrophosphate depletion, embryonic lethality, CNS malformations, and anemia.* Proc Natl Acad Sci U S A, 2006. **103**(43): p. 15927-32.

13. Huizing, M., et al., *Cloning of the human carnitine-acylcarnitine carrier cDNA and identification of the molecular defect in a patient.* Am J Hum Genet, 1997. **61**(6): p. 1239-45.

14. Palmieri, L., et al., *Identification in Saccharomyces cerevisiae of two isoforms of a novel mitochondrial transporter for 2-oxoadipate and 2-oxoglutarate.* J Biol Chem, 2001. **276**(3): p. 1916-22.

15. Fiermonte, G., et al., *Identification of the mitochondrial ATP-Mg/Pi transporter. Bacterial expression, reconstitution, functional characterization, and tissue distribution.* J Biol Chem, 2004. **279**(29): p. 30722-30.

16. Marobbio, C.M., et al., *Identification and functional reconstitution of yeast mitochondrial carrier for S-adenosylmethionine.* EMBO J, 2003. **22**(22): p. 5975-82.

17. Shaw, G.C., et al., *Mitoferrin is essential for erythroid iron assimilation.* Nature, 2006. **440**(7080): p. 96-100.

18. Sekoguchi, E., et al., *A novel mitochondrial carnitine-acylcarnitine translocase induced by partial hepatectomy and fasting.* J Biol Chem, 2003. **278**(40): p. 38796-802.

19. Titus, S.A. and R.G. Moran, *Retrovirally mediated complementation of the glyB phenotype. Cloning of a human gene encoding the carrier for entry of folates into mitochondria.* J Biol Chem, 2000. **275**(47): p. 36811-7.

20. Floyd, S., et al., *The insulin-like growth factor-I-mTOR signaling pathway induces the mitochondrial pyrimidine nucleotide carrier to promote cell growth.* Mol Biol Cell, 2007. **18**(9): p. 3545-55.

21. Guernsey, D.L., et al., *Mutations in mitochondrial carrier family gene SLC25A38 cause nonsyndromic autosomal recessive congenital sideroblastic anemia.* Nat Genet, 2009. **41**(6): p. 651-3.

22. Traba, J., J. Satrustegui, and A. del Arco, *Characterization of SCaMC-3-like/slc25a41, a novel calcium-independent mitochondrial ATP-Mg/Pi carrier.* Biochem J, 2009. **418**(1): p. 125-33.

23. Fiermonte, G., et al., *A novel member of solute carrier family 25 (SLC25A42) is a transporter of coenzyme A and adenosine 3',5'-diphosphate in human mitochondria.* J Biol Chem, 2009. **284**(27): p. 18152-9.

24. Lamarca, V., et al., *Exposure of any of two proapoptotic domains of presenilin 1-associated protein/mitochondrial carrier homolog 1 on the surface of mitochondria is sufficient for induction of apoptosis in a Bax/Bak-independent manner.* Eur J Cell Biol, 2008. **87**(5): p. 325-34.

25. Robinson, A.J., E.R. Kunji, and A. Gross, *Mitochondrial carrier homolog 2 (MTCH2): the recruitment and evolution of a mitochondrial carrier protein to a critical player in apoptosis.* Exp Cell Res, 2012. **318**(11): p. 1316-23.

26. Luongo, T.S., et al., *SLC25A51 is a mammalian mitochondrial NAD(+) transporter.* Nature, 2020. **588**(7836): p. 174-179.
