# Supplementary material for: Comprehensive Analysis and Validation of Solute Carrier Family 25 (SLC25) and Its Correlation with Immune Infiltration in Pan-Cancer
Source: Biomed Res Int. 2022 Oct 8;2022:4009354. doi: 10.1155/2022/4009354 (PMC9569204; doi:10.1155/2022/4009354)
Supplement: Supplementary Materials — Table S1: the genes of SLC25 family and its references. Table S2: the abbreviation of 33 cancer types. Table S3: the information of primer sequences. Table S4: the correlation of SLC25A4&SLC25A7 expression and clinical pathological parameters in gastric cancer. Table S5: the correlation of SLC25A23&SLC25A7 expression and clinical pathological parameters in colon cancer. Table S6: the original data for the association between the expression of SLC25A4 and the clinicopathological parameters of gastric cancer specimens. Table S7: the original data for the association between the expression of SLC25A7 and the clinicopathological parameters of gastric cancer specimens. Table S8: the original data for the association between the expression of SLC25A7 and the clinicopathological parameters of colon cancer specimens. Table S9: the original data for the association between the expression of SLC25A23 and the clinicopathological parameters of colon cancer specimens. Figure S1: the differential expression of other genes of SLC25 family. Figure S1 legend. The legend of Figure S1. [file 4009354.f1.zip › Table S3 (1).docx]

| **Table S3.The information of primer sequences.** | |  |
| --- | --- | --- |
| **Gene** | **Fwd Sequence(5' to 3')** | **Rev Sequence(5' to 3')** |
| β-actin | ATGTGGCCGAGGACTTTGATT | AGTGGGGTGGCTTTTAGGATG |
| SLC25A4 | TTGTGAGCTGGATGATTGCC | TGTACATAATATCGGCCCCTTTC |
| SLC25A7 | AGTTCCTCACCGCAGGGAAAGA | GTAGCGAGGTTTGATTCCGTGG |
| SLC25A23 | GGCCAGATAGCCAGTTACCC | GGGACAGGATGTGACGTAGC |
