# Supplementary material for: Comprehensive Analysis and Validation of Solute Carrier Family 25 (SLC25) and Its Correlation with Immune Infiltration in Pan-Cancer
Source: Biomed Res Int. 2022 Oct 8;2022:4009354. doi: 10.1155/2022/4009354 (PMC9569204; doi:10.1155/2022/4009354)
Supplement: Supplementary Materials — Table S1: the genes of SLC25 family and its references. Table S2: the abbreviation of 33 cancer types. Table S3: the information of primer sequences. Table S4: the correlation of SLC25A4&SLC25A7 expression and clinical pathological parameters in gastric cancer. Table S5: the correlation of SLC25A23&SLC25A7 expression and clinical pathological parameters in colon cancer. Table S6: the original data for the association between the expression of SLC25A4 and the clinicopathological parameters of gastric cancer specimens. Table S7: the original data for the association between the expression of SLC25A7 and the clinicopathological parameters of gastric cancer specimens. Table S8: the original data for the association between the expression of SLC25A7 and the clinicopathological parameters of colon cancer specimens. Table S9: the original data for the association between the expression of SLC25A23 and the clinicopathological parameters of colon cancer specimens. Figure S1: the differential expression of other genes of SLC25 family. Figure S1 legend. The legend of Figure S1. [file 4009354.f1.zip › Table S4 (1).docx]

| **Table S4 The correlation of SLC25A4&SLC25A7 expression and clinical pathological parameters in gastric cancer** | | | | | | | | |
| --- | --- | --- | --- | --- | --- | --- | --- | --- |
| Variables | Cases | SLC25A4 | | *P* | Cases | SLC25A7 | | *P* |
|  |  | High(%) | Low(%) |  |  | High(%) | Low(%) |  |
| Gender | 23 |  |  | 0.371 | 23 |  |  | 1.000 |
| Male | 16 | 7(43.8) | 9(56.2) |  | 15 | 7(46.7) | 8(53.3) |  |
| Female | 7 | 4(57.1) | 3(42.9) |  | 8 | 4(50.0) | 4(50.0) |  |
| Age(years) | 23 |  |  | 0.220 | 23 |  |  | 1.000 |
| >60 | 12 | 8(66.7) | 4(33.3) |  | 13 | 6(46.2) | 7(53.8) |  |
| <=60 | 11 | 4(36.4) | 7(63.6) |  | 10 | 5(50.0) | 5(50.0) |  |
| Smoking | 23 |  |  | 0.537 | 23 |  |  | 0.856 |
| Yes | 6 | 2(33.3) | 4(66.7) |  | 4 | 2(50.0) | 2(50.0) |  |
| No | 15 | 8(53.3) | 7(46.7) |  | 12 | 5(41.7) | 7(58.3) |  |
| Missing | 2 |  |  |  | 7 |  |  |  |
| Drinking | 23 |  |  | 0.640 | 23 |  |  | 0.856 |
| Yes | 4 | 1(25.0) | 3(75.0) |  | 4 | 2(50.0) | 2(50.0) |  |
| No | 17 | 10(58.8) | 7(41.2) |  | 11 | 5(45.5) | 6(54.4) |  |
| Missing | 2 |  |  |  | 8 |  |  |  |
| Lauren classification | 23 |  |  | 0.250 | 23 |  |  | 0.600 |
| Diffuse | 14 | 5(35.7) | 9(64.3) |  | 14 | 6(42.9) | 8(57.1) |  |
| Mixed | 3 | 2(66.7) | 1(33.3) |  | 3 | 1(33.3) | 2(66.7) |  |
| Intestinal | 4 | 3(66.7) | 1(33.3) |  | 6 | 4(66.7) | 2(33.3) |  |
| Missing | 2 |  |  |  | 0 |  |  |  |
| Depth of invasion | 23 |  |  | 0.695 | 23 |  |  | 0.676 |
| Serosal layer or invasion adjacent organs | 13 | 6(46.2) | 7(53.8)) |  | 13 | 7(53.8) | 6(46.2) |  |
| Mucous and submucosal layer | 2 | 2(100.0) | 0(0.0) |  | 2 | 0(0.0) | 2(100.0) |  |
| Muscular and subserosa layer | 4 | 2(50.0) | 2(50.0) |  | 3 | 2(66.7) | 1(33.3) |  |
| Missing | 4 |  |  |  | 5 |  |  |  |
| Lymph node metastasis | 23 |  |  | 1.000 | 23 |  |  | 1.000 |
| Positive | 14 | 7(50.0) | 7(50.0) |  | 15 | 7(46.7) | 8(53.3) |  |
| Negative | 7 | 4(57.1) | 3(42.9) |  | 8 | 4(66.7) | 4 |  |
| Missing | 2 |  |  |  | 0 |  |  |  |
| TNMstage | 23 |  |  | 0.107 | 23 |  |  | 1.000 |
| I+II | 9 | 6(66.7) | 3(33.3) |  | 10 | 5(50.0) | 5(50.0) |  |
| III+IV | 12 | 4(33.3) | 8(66.7) |  | 12 | 6(50.0) | 6(50.0) |  |
| Missing | 2 |  |  |  | 1 |  |  |  |
