# Supplementary material for: Comprehensive Analysis and Validation of Solute Carrier Family 25 (SLC25) and Its Correlation with Immune Infiltration in Pan-Cancer
Source: Biomed Res Int. 2022 Oct 8;2022:4009354. doi: 10.1155/2022/4009354 (PMC9569204; doi:10.1155/2022/4009354)
Supplement: Supplementary Materials — Table S1: the genes of SLC25 family and its references. Table S2: the abbreviation of 33 cancer types. Table S3: the information of primer sequences. Table S4: the correlation of SLC25A4&SLC25A7 expression and clinical pathological parameters in gastric cancer. Table S5: the correlation of SLC25A23&SLC25A7 expression and clinical pathological parameters in colon cancer. Table S6: the original data for the association between the expression of SLC25A4 and the clinicopathological parameters of gastric cancer specimens. Table S7: the original data for the association between the expression of SLC25A7 and the clinicopathological parameters of gastric cancer specimens. Table S8: the original data for the association between the expression of SLC25A7 and the clinicopathological parameters of colon cancer specimens. Table S9: the original data for the association between the expression of SLC25A23 and the clinicopathological parameters of colon cancer specimens. Figure S1: the differential expression of other genes of SLC25 family. Figure S1 legend. The legend of Figure S1. [file 4009354.f1.zip › Table S5 (1).docx]

| **Table S5 The correlation of SLC25A23&SLC25A7 expression and clinical pathological parameters in colon cancer** | | | | | | | | |
| --- | --- | --- | --- | --- | --- | --- | --- | --- |
| Variables | Cases | SLC25A23 | | *P* | Cases | SLC25A7 |  | *P* |
|  |  | High(%) | Low(%) |  |  | High(%) | Low(%) |  |
| Gender | 29 |  |  | 0.466 | 8 |  |  | 1.000 |
| Male | 15 | 9(60) | 6(40) |  | 2 | 1(50.0) | 1(50.0) |  |
| Female | 14 | 6(42.9) | 8(57.1) |  | 6 | 3(50.0) | 3(50.0) |  |
| Age(years) | 29 |  |  | 1.000 | 8 |  |  | 1.000 |
| >60 | 9 | 5(55.6) | 4(44.4) |  | 1 | 0(0.0) | 1(100.0) |  |
| <=60 | 20 | 10(50.0) | 10(50.0) |  | 7 | 4(57.1) | 3(42.9) |  |
| Smoking | 29 |  |  | 0.682 | 8 |  |  | 1.000 |
| Yes | 8 | 5(62.5) | 3(37.5) |  | 2 | 1(50.0) | 1(50.0) |  |
| No | 21 | 10(47.6) | 11(62.4) |  | 6 | 3(50.0) | 3(50.0) |  |
| Drinking | 29 |  |  | 1.000 | 8 |  |  | 1.000 |
| Yes | 7 | 4(57.1) | 3(42.9) |  | 1 | 1(100.0) | 0(0.0) |  |
| No | 22 | 11(50.0) | 11(50.0) |  | 7 | 3(42.9) | 4(57.1) |  |
| Family history | 29 |  |  | 0.224 | 8 |  |  | 1.000 |
| Yes | 2 | 0(0.0) | 2(100.0) |  | 1 | 4(57.1) | 3(42.9) |  |
| No | 27 | 15(55.6) | 12(44.4) |  | 7 | 0(0.0) | 1(100.0) |  |
| Maximum diameter (cm) | 29 |  |  | 0.318 | 8 |  |  | 1.000 |
| >4.75 | 29 | 15(51.7) | 14(48.3) |  | 8 | 4(50.0) | 4(50.0) |  |
| <=4.75 | 0 | 0(0.0) | 0(0.0) |  | 0 | 0(0.0) | 0(0.0) |  |
| Differentiation degree | 29 |  |  | 0.710 | 8 |  |  | 0.486 |
| Well/moderate | 18 | 10(55.6) | 8(44.4) |  | 4 | 1(25.0) | 3(75.0) |  |
| Poor/mucinous | 11 | 5(45.5) | 6(54.5) |  | 4 | 3(75.0) | 1(25.0) |  |
| Growth pattern | 29 |  |  | 0.462 | 8 |  |  | 0.455 |
| Infiltrative | 16 | 7(43.8) | 9(26.2) |  | 4 | 3(75.0) | 1(25.0) |  |
| Nested/cloddy | 13 | 8(61.5) | 5(38.5) |  | 4 | 1(25.0) | 3(75.0) |  |
| Lymphatic/venous invasion | 29 |  |  | 0.330 | 8 |  |  | 0.429 |
| Yes | 4 | 1(25.0) | 3(75.0) |  | 2 | 2(100.0) | 0(0.0) |  |
| No | 25 | 14(56.0) | 11(44.0) |  | 6 | 2(33.3) | 4(66.7) |  |
| Invasive extent | 29 |  |  | 0.450 | 8 |  |  | 1.000 |
| T1-2 | 11 | 7(63.6) | 4(36.4) |  | 2 | 1(50.0) | 1(50.0) |  |
| T3-4 | 18 | 8(44.4) | 10(55.6) |  | 6 | 3(50.0) | 3(50.0) |  |
| Tumor location | 29 |  |  | 0.894 | 8 |  |  | 0.429 |
| Rectum | 23 | 12(52.2) | 11(47.8) |  | 6 | 4(66.7) | 2(33.3) |  |
| Colon | 6 | 3(50.0) | 3(50.0) |  | 2 | 0(0.0) | 2(100.0) |  |
| Lymph node metastasis | 29 |  |  | 0.139 | 8 |  |  | 1.000 |
| Positive | 12 | 4(33.3) | 8(66.7) |  | 4 | 3(75.0) | 1(25.0) |  |
| Negative | 17 | 11(64.7) | 6(35.3) |  | 4 | 1(25.0) | 3(75.0) |  |
| TNMstage | 29 |  |  | 0.066 | 8 |  |  | 1.000 |
| I+II | 16 | 11(68.8) | 5(31.2) |  | 2 | 1(50.0) | 1(50.0) |  |
| III+IV | 13 | 4(30.1) | 9(69.9) |  | 6 | 3(50.0) | 3(50.0) |  |
