# Supplementary material for: Comprehensive Analysis and Validation of Solute Carrier Family 25 (SLC25) and Its Correlation with Immune Infiltration in Pan-Cancer
Source: Biomed Res Int. 2022 Oct 8;2022:4009354. doi: 10.1155/2022/4009354 (PMC9569204; doi:10.1155/2022/4009354)
Supplement: Supplementary Materials — Table S1: the genes of SLC25 family and its references. Table S2: the abbreviation of 33 cancer types. Table S3: the information of primer sequences. Table S4: the correlation of SLC25A4&SLC25A7 expression and clinical pathological parameters in gastric cancer. Table S5: the correlation of SLC25A23&SLC25A7 expression and clinical pathological parameters in colon cancer. Table S6: the original data for the association between the expression of SLC25A4 and the clinicopathological parameters of gastric cancer specimens. Table S7: the original data for the association between the expression of SLC25A7 and the clinicopathological parameters of gastric cancer specimens. Table S8: the original data for the association between the expression of SLC25A7 and the clinicopathological parameters of colon cancer specimens. Table S9: the original data for the association between the expression of SLC25A23 and the clinicopathological parameters of colon cancer specimens. Figure S1: the differential expression of other genes of SLC25 family. Figure S1 legend. The legend of Figure S1. [file 4009354.f1.zip › Table S6 (1).docx]

| **Table S6. The original data for the association between the expression of SLC25A4 and the clinicopathological parameters of gastric cancer specimens.** | | | | | | | | | | | |
| --- | --- | --- | --- | --- | --- | --- | --- | --- | --- | --- | --- |
| **Sample ID** | **Cancer-2^-ΔCT** | **Normal-2^-ΔCT** | **Expression of SLC25A4** | **Gender** | **Age(years)** | **Smoking** | **Drinking** | **Lauren classification** | **Depth of invasion** | **Lymph node metastasis** | **TNM stage** |
| Q11 | 1E-04 | 0.00147 | High | Female | <60 | NA | no | NA | NA | yes | NA |
| Q12 | 0.0073 | 0.001178 | High | Female | >=60 | no | no | NA | NA | yes | NA |
| Q20 | 0.0043 | 0.011203 | High | Male | >=60 | yes | no | mixed | sm | NA | I+II |
| Q24 | 0.0035 | 0.008669 | High | Male | >=60 | no | no | mixed | se | no | I+II |
| Q25 | 0.0004 | 0.017701 | Low | Male | <60 | no | no | diffuse | se | yes | III+IV |
| YS22 | 0.0004 | 0.018841 | Low | Male | >=60 | NA | NA | diffuse | NA | yes | III+IV |
| YS30 | 0.0016 | 0.043889 | Low | Male | <60 | no | no | diffuse | NA | NA | III+IV |
| YS37 | 0.001 | 0.009685 | Low | Female | >=60 | no | no | diffuse | se | no | I+II |
| YS48 | 0.0041 | 0.04095 | High | Male | >=60 | NA | NA | intestinal | se | yes | III+IV |
| YS50 | 0.0021 | 0.024861 | Low | Male | <60 | yes | no | mixed | se | yes | III+IV |
| YS51 | 0.0139 | 0.045123 | High | Female | >=60 | no | no | diffuse | se | no | I+II |
| YS52 | 0.0028 | 0.016402 | High | Male | >=60 | no | no | intestinal | sm | no | I+II |
| YS54 | 0.0042 | 0.031686 | High | Male | <60 | no | no | diffuse | se | yes | III+IV |
| YS55 | 0.0011 | 0.007442 | Low | Male | <60 | no | yes | diffuse | se | yes | III+IV |
| YS56 | 0.0016 | 0.005719 | Low | Male | <60 | yes | no | diffuse | se | yes | III+IV |
| YS57 | 4E-05 | 0.002197 | Low | Male | >=60 | yes | yes | diffuse | ss | no | I+II |
| YS59 | 0.0073 | 0.005962 | High | Male | >=60 | no | no | diffuse | se | yes | III+IV |
| YS60 | 0.001 | 0.015953 | Low | Male | <60 | yes | yes | diffuse | ss | no | I+II |
| YS62 | 0.0173 | 0.002259 | High | Male | >=60 | yes | yes | intestinal | ss | yes | I+II |
| YS63 | 0.0043 | 0.003961 | High | Female | <60 | no | no | diffuse | se | yes | III+IV |
| YS64 | 0.0026 | 0.004275 | High | Female | <60 | no | no | diffuse | ss | no | I+II |
| YS65 | 0.0021 | 0.010672 | Low | Male | >=60 | no | no | intestinal | se | yes | III+IV |
| YS66 | 0.0011 | 0.004129 | Low | Female | <60 | no | no | diffuse | se | yes | III+IV |
