# Supplementary material for: Comprehensive Analysis and Validation of Solute Carrier Family 25 (SLC25) and Its Correlation with Immune Infiltration in Pan-Cancer
Source: Biomed Res Int. 2022 Oct 8;2022:4009354. doi: 10.1155/2022/4009354 (PMC9569204; doi:10.1155/2022/4009354)
Supplement: Supplementary Materials — Table S1: the genes of SLC25 family and its references. Table S2: the abbreviation of 33 cancer types. Table S3: the information of primer sequences. Table S4: the correlation of SLC25A4&SLC25A7 expression and clinical pathological parameters in gastric cancer. Table S5: the correlation of SLC25A23&SLC25A7 expression and clinical pathological parameters in colon cancer. Table S6: the original data for the association between the expression of SLC25A4 and the clinicopathological parameters of gastric cancer specimens. Table S7: the original data for the association between the expression of SLC25A7 and the clinicopathological parameters of gastric cancer specimens. Table S8: the original data for the association between the expression of SLC25A7 and the clinicopathological parameters of colon cancer specimens. Table S9: the original data for the association between the expression of SLC25A23 and the clinicopathological parameters of colon cancer specimens. Figure S1: the differential expression of other genes of SLC25 family. Figure S1 legend. The legend of Figure S1. [file 4009354.f1.zip › Table S7 (1).docx]

| **Table S7. The original data for the association between the expression of SLC25A7 and the clinicopathological parameters of gastric cancer specimens.** | | | | | | | | | | | |
| --- | --- | --- | --- | --- | --- | --- | --- | --- | --- | --- | --- |
| **Sample ID** | **Cancer-2^-ΔCT** | **Normal-2^-ΔCT** | **Expression of SLC25A7** | **Gender** | **Age(years)** | **Smoking** | **Drinking** | **Lauren classification** | **Depth of invasion** | **Lymph node metastasis** | **TNM stage** |
| Q15 | 4.26E-05 | NA | High | Male | >=60 | NA | NA | diffuse | se | yes | NA |
| Q17 | 0.170755 | NA | High | Female | <60 | no | no | diffuse | se | yes | III+IV |
| Q18 | 0.000725 | 6.97E-06 | High | Male | <60 | no | no | diffuse | NA | yes | III+IV |
| Q24 | 1.88E-05 | 2.59E-06 | Low | Male | >=60 | no | no | mixed | se | no | I+II |
| Q25 | 0.000173 | 3.55E-05 | High | Male | <60 | no | no | diffuse | se | yes | III+IV |
| Q28 | 3.71E-06 | 8.18E-06 | Low | Male | >=60 | no | no | diffuse | NA | no | NA |
| Q34 | 4.09E-06 | NA | Low | Male | >=60 | no | no | intestinal | se | yes | III+IV |
| Q37 | 8.58E-06 | NA | Low | Male | <60 | no | yes | diffuse | se | yes | III+IV |
| Q40 | 2.15E-06 | NA | Low | Female | >=60 | no | no | intestinal | sm | yes | I+II |
| Q43 | 5.77E-05 | 2.19E-06 | High | Male | >=60 | yes | yes | intestinal | se | yes | III+IV |
| Q48 | 4.89E-05 | 1.08E-05 | High | Male | >=60 | NA | NA | mixed | NA | no | I+II |
| Q54 | 0.023683 | 4.15E-06 | High | Female | >=60 | NA | NA | intestinal | se | no | I+II |
| Q58 | 2.36E-05 | 5.59E-06 | Low | Female | >=60 | NA | NA | diffuse | NA | yes | III+IV |
| Q72 | 5.73E-05 | NA | High | Female | <60 | NA | NA | diffuse | se | no | I+II |
| YS19 | 3.23E-06 | NA | Low | Female | >=60 | NA | NA | diffuse | ss | yes | I+II |
| YS41 | 9.86E-06 | NA | Low | Male | >=60 | yes | no | diffuse | sm | no | I+II |
| YS50 | 1.09E-05 | NA | Low | Male | <60 | yes | no | mixed | se | yes | III+IV |
| YS51 | 3.58E-05 | NA | High | Female | >=60 | no | no | diffuse | se | no | I+II |
| YS55 | 3.27E-05 | NA | Low | Male | <60 | no | yes | diffuse | se | yes | III+IV |
| YS58 | 3.51E-05 | NA | High | Male | <60 | no | no | intestinal | ss | yes | III+IV |
| YS6 | 7.64E-07 | 9.47E-07 | Low | Male | <60 | NA | NA | diffuse | NA | no | I+II |
| YS62 | 0.000105 | NA | High | Male | >=60 | yes | yes | intestinal | ss | yes | I+II |
| YS66 | 2.55E-05 | 9.32E-05 | Low | Female | <60 | no | no | diffuse | se | yes | III+IV |
