# Supplementary material for: Comprehensive Analysis and Validation of Solute Carrier Family 25 (SLC25) and Its Correlation with Immune Infiltration in Pan-Cancer
Source: Biomed Res Int. 2022 Oct 8;2022:4009354. doi: 10.1155/2022/4009354 (PMC9569204; doi:10.1155/2022/4009354)
Supplement: Supplementary Materials — Table S1: the genes of SLC25 family and its references. Table S2: the abbreviation of 33 cancer types. Table S3: the information of primer sequences. Table S4: the correlation of SLC25A4&SLC25A7 expression and clinical pathological parameters in gastric cancer. Table S5: the correlation of SLC25A23&SLC25A7 expression and clinical pathological parameters in colon cancer. Table S6: the original data for the association between the expression of SLC25A4 and the clinicopathological parameters of gastric cancer specimens. Table S7: the original data for the association between the expression of SLC25A7 and the clinicopathological parameters of gastric cancer specimens. Table S8: the original data for the association between the expression of SLC25A7 and the clinicopathological parameters of colon cancer specimens. Table S9: the original data for the association between the expression of SLC25A23 and the clinicopathological parameters of colon cancer specimens. Figure S1: the differential expression of other genes of SLC25 family. Figure S1 legend. The legend of Figure S1. [file 4009354.f1.zip › Table S8 (1).docx]

| **Table S8. The original data for the association between the expression of SLC25A7 and the clinicopathological parameters of colon cancer specimens.** | | | | | | | | | | | | | | | | |
| --- | --- | --- | --- | --- | --- | --- | --- | --- | --- | --- | --- | --- | --- | --- | --- | --- |
| **Sample ID** | **Cancer-2^-ΔCT** | **Normal-2^-ΔCT** | **Expression of SLC25A7** | **Gender** | **Age(years)** | **Smoking** | **Drinking** | **Family history** | **Maximum diameter (cm)** | **Lymph Node metastasis** | **Differentiation degree** | **Growth pattern** | **Lymphatic/venous invasion** | **Invasive extent** | **TNM stage** | **Tumor location** |
| Y113 | 2.97E-05 | 5.463E-05 | Low | Female | <60 | no | no | no | <=4.75 | yes | well | Nested/cloddy | no | T3-4 | III+IV | rectum |
| Y17 | 0.000185 | NA | High | Male | >=60 | yes | yes | yes | <=4.75 | yes | poor | Infiltrative | no | T3-4 | III+IV | rectum |
| Y33 | 4.8E-06 | NA | Low | Female | >=60 | no | no | yes | <=4.75 | yes | well | Nested/cloddy | no | T1-2 | III+IV | colon |
| Y5 | 3.41E-05 | 5.896E-05 | Low | Male | >=60 | yes | no | yes | <=4.75 | no | poor | Nested/cloddy | no | T3-4 | I+II | rectum |
| Y62 | 2.09E-06 | 1.681E-05 | Low | Female | >=60 | no | no | yes | <=4.75 | yes | well | Infiltrative | no | T3-4 | III+IV | colon |
| Y77 | 0.000362 | NA | High | Female | >=60 | no | no | yes | <=4.75 | no | poor | Infiltrative | yes | T3-4 | I+II | rectum |
| Y79 | 0.000338 | NA | High | Female | >=60 | no | no | yes | <=4.75 | yes | well | Nested/cloddy | no | T1-2 | III+IV | rectum |
| Y86 | 0.001554 | NA | High | Female | >=60 | no | no | yes | <=4.75 | yes | poor | Infiltrative | yes | T3-4 | III+IV | rectum |
