# Supplementary material for: Comprehensive Analysis and Validation of Solute Carrier Family 25 (SLC25) and Its Correlation with Immune Infiltration in Pan-Cancer
Source: Biomed Res Int. 2022 Oct 8;2022:4009354. doi: 10.1155/2022/4009354 (PMC9569204; doi:10.1155/2022/4009354)
Supplement: Supplementary Materials — Table S1: the genes of SLC25 family and its references. Table S2: the abbreviation of 33 cancer types. Table S3: the information of primer sequences. Table S4: the correlation of SLC25A4&SLC25A7 expression and clinical pathological parameters in gastric cancer. Table S5: the correlation of SLC25A23&SLC25A7 expression and clinical pathological parameters in colon cancer. Table S6: the original data for the association between the expression of SLC25A4 and the clinicopathological parameters of gastric cancer specimens. Table S7: the original data for the association between the expression of SLC25A7 and the clinicopathological parameters of gastric cancer specimens. Table S8: the original data for the association between the expression of SLC25A7 and the clinicopathological parameters of colon cancer specimens. Table S9: the original data for the association between the expression of SLC25A23 and the clinicopathological parameters of colon cancer specimens. Figure S1: the differential expression of other genes of SLC25 family. Figure S1 legend. The legend of Figure S1. [file 4009354.f1.zip › Table S9 (1).docx]

| **Table S9. The original data for the association between the expression of SLC25A23 and the clinicopathological parameters of colon cancer specimens.** | | | | | | | | | | | | | | | | |
| --- | --- | --- | --- | --- | --- | --- | --- | --- | --- | --- | --- | --- | --- | --- | --- | --- |
| **Sample ID** | **Cancer-2^-ΔCT** | **Normal-2^-ΔCT** | **Expression of SLC25A7** | **Gender** | **Age(years)** | **Smoking** | **Drinking** | **Family history** | **Maximum diameter (cm)** | **Lymph Node metastasis** | **Differentiation degree** | **Growth pattern** | **Lymphatic/venous invasion** | **Invasive extent** | **TNM stage** | **Tumor location** |
| Y102 | 0.01951 | 0.0298 | High | Male | >=60 | no | no | no | >4.75 | no | well | Infiltrative | no | T3-4 | I+II | rectum |
| Y116 | 0.03955 | 0.1199 | High | Female | <60 | no | no | no | >4.75 | no | poor | Infiltrative | no | T3-4 | I+II | rectum |
| Y121 | 0.03955 | 0.0544 | High | Male | >=60 | yes | no | no | >4.75 | no | well | Infiltrative | no | T1-2 | I+II | rectum |
| Y124 | 0.00168 | 0.0124 | Low | Female | >=60 | no | no | no | >4.75 | yes | well | Infiltrative | no | T3-4 | III+IV | rectum |
| Y126 | 0.00282 | 0.0046 | Low | Female | <60 | no | no | no | >4.75 | no | poor | Infiltrative | no | T1-2 | I+II | rectum |
| Y134 | 0.00962 | 0.0177 | High | Female | <60 | yes | no | no | >4.75 | yes | poor | Nested/cloddy | no | T1-2 | III+IV | rectum |
| Y17 | 0.0007 | 0.0219 | Low | Male | >=60 | yes | yes | no | >4.75 | yes | poor | Infiltrative | no | T3-4 | III+IV | rectum |
| Y18 | 0.00537 | 0.0028 | Low | Female | <60 | no | no | no | >4.75 | yes | poor | Nested/cloddy | no | T3-4 | III+IV | rectum |
| Y19 | 0.04299 | 0.0407 | High | Male | >=60 | no | no | no | >4.75 | no | well | Infiltrative | no | T3-4 | I+II | rectum |
| Y21 | 0.00416 | 0.0148 | Low | Female | >=60 | no | no | no | >4.75 | no | well | Infiltrative | yes | T1-2 | I+II | rectum |
| Y23 | 0.0091 | 0.0181 | High | Male | <60 | no | yes | no | >4.75 | yes | well | Nested/cloddy | no | T3-4 | III+IV | colon |
| Y29 | 0.00167 | 0.0097 | Low | Male | >=60 | no | no | no | >4.75 | no | poor | Infiltrative | no | T3-4 | III+IV | colon |
| Y30 | 0.01201 | 0.0199 | High | Female | >=60 | no | no | no | >4.75 | no | well | Nested/cloddy | no | T1-2 | I+II | rectum |
| Y32 | 0.05403 | 0.0163 | High | Male | >=60 | no | yes | no | >4.75 | yes | well | Nested/cloddy | no | T1-2 | III+IV | rectum |
| Y33 | 0.00929 | 0.0213 | High | Female | >=60 | no | no | no | >4.75 | yes | well | Nested/cloddy | no | T1-2 | III+IV | colon |
| Y38 | 0.01964 | 0.0242 | High | Male | >=60 | yes | yes | no | >4.75 | no | well | Nested/cloddy | no | T3-4 | I+II | rectum |
| Y48 | 0.00719 | 0.0179 | Low | Female | <60 | no | no | no | >4.75 | no | well | Nested/cloddy | no | T1-2 | I+II | rectum |
| Y5 | 0.00942 | 0.003 | High | Male | >=60 | yes | no | no | >4.75 | no | poor | Nested/cloddy | no | T3-4 | I+II | rectum |
| Y55 | 0.01448 | 0.0097 | High | Male | >=60 | no | no | no | >4.75 | no | well | Infiltrative | no | T1-2 | I+II | rectum |
| Y62 | 0.00534 | 0.0075 | Low | Female | >=60 | no | no | no | >4.75 | yes | well | Infiltrative | no | T3-4 | III+IV | colon |
| Y63 | 0.02164 | 0.0292 | High | Female | <60 | no | no | no | >4.75 | no | well | Nested/cloddy | no | T3-4 | I+II | colon |
| Y77 | 0.00809 | 0.0109 | High | Female | >=60 | no | no | no | >4.75 | no | poor | Infiltrative | yes | T3-4 | I+II | rectum |
| Y79 | 0.00592 | 0.0548 | Low | Female | >=60 | no | no | no | >4.75 | yes | well | Nested/cloddy | no | T1-2 | III+IV | rectum |
| Y84 | 0.00568 | 0.0038 | Low | Male | >=60 | yes | no | no | >4.75 | yes | well | Nested/cloddy | yes | T3-4 | III+IV | rectum |
| Y86 | 0.00399 | 0.0202 | Low | Female | >=60 | no | no | no | >4.75 | yes | poor | Infiltrative | yes | T3-4 | III+IV | rectum |
| Y89 | 0.0076 | 0.0125 | High | Male | <60 | yes | yes | no | >4.75 | no | poor | Infiltrative | no | T1-2 | I+II | rectum |
| Y91 | 0.00359 | 0.0128 | Low | Male | <60 | yes | yes | yes | >4.75 | no | well | Nested/cloddy | no | T3-4 | I+II | colon |
| Y94 | 0.0019 | 0.0205 | Low | Male | >=60 | no | no | yes | >4.75 | no | well | Infiltrative | no | T3-4 | I+II | rectum |
| Y95 | 0.00383 | 0.0157 | Low | Male | >=60 | no | yes | no | >4.75 | yes | poor | Infiltrative | no | T3-4 | III+IV | rectum |
